# Use of Clinical Data Interchange Standards Consortium (CDISC) Standards for Real-world Data: Expert Perspectives From a Qualitative Delphi Survey

**DOI:** 10.2196/30363

**Published:** 2022-01-27

**Authors:** Rhonda Facile, Erin Elizabeth Muhlbradt, Mengchun Gong, Qingna Li, Vaishali Popat, Frank Pétavy, Ronald Cornet, Yaoping Ruan, Daisuke Koide, Toshiki I Saito, Sam Hume, Frank Rockhold, Wenjun Bao, Sue Dubman, Barbara Jauregui Wurst

**Affiliations:** 1 Clinical Data Interchange Standards Consortium Austin, TX United States; 2 Medical Science and Computing, a Guidehouse company Rockville, MD United States; 3 Digital Health China Technologies Bejing China; 4 Institute of Health Management Southern Medical University Guangzhou China; 5 Institute of Clinical Pharmacology Xiyuan Hospital of China Academy of Chinese Medical Sciences Beijing China; 6 Key Laboratory for Clinical Research and Evaluation of Traditional Chinese Medicine of National Medical Products Administration Beijing China; 7 National Clinical Research Center for Chinese Medicine Cardiology Beijing China; 8 Food and Drug Administration Center for Drug Evaluation Research Silver Spring, MD United States; 9 European Medicines Agency Amsterdam Netherlands; 10 Department of Medical Informatics Amsterdam Public Health Research Institute Amsterdam University Medical Centers - University of Amsterdam Amsterdam Netherlands; 11 LinkDoc Technologies Beijing China; 12 Department of Biostatistics & Bioinformatics Graduate School of Medicine University of Tokyo Tokyo Japan; 13 National Hospital Organization Nagoya Medical Center Nagoya Japan; 14 Duke Clinical Research Institute Duke University Medical Center Durham, NC United States; 15 JMP Life Sciences SAS Institute Inc Cary, NC United States

**Keywords:** real-world data, real-world evidence, clinical trials, Delphi survey, clinical data standards, regulatory submission, academic research, public health data, registry data, electronic health records, observational data, data integration, FAIR principles

## Abstract

**Background:**

Real-world data (RWD) and real-world evidence (RWE) are playing increasingly important roles in clinical research and health care decision-making. To leverage RWD and generate reliable RWE, data should be well defined and structured in a way that is semantically interoperable and consistent across stakeholders. The adoption of data standards is one of the cornerstones supporting high-quality evidence for the development of clinical medicine and therapeutics. Clinical Data Interchange Standards Consortium (CDISC) data standards are mature, globally recognized, and heavily used by the pharmaceutical industry for regulatory submissions. The CDISC RWD Connect Initiative aims to better understand the barriers to implementing CDISC standards for RWD and to identify the tools and guidance needed to more easily implement them.

**Objective:**

The aim of this study is to understand the barriers to implementing CDISC standards for RWD and to identify the tools and guidance that may be needed to implement CDISC standards more easily for this purpose.

**Methods:**

We conducted a qualitative Delphi survey involving an expert advisory board with multiple key stakeholders, with 3 rounds of input and review.

**Results:**

Overall, 66 experts participated in round 1, 56 in round 2, and 49 in round 3 of the Delphi survey. Their inputs were collected and analyzed, culminating in group statements. It was widely agreed that the standardization of RWD is highly necessary, and the primary focus should be on its ability to improve data sharing and the quality of RWE. The priorities for RWD standardization included electronic health records, such as data shared using Health Level 7 Fast Health care Interoperability Resources (FHIR), and the data stemming from observational studies. With different standardization efforts already underway in these areas, a gap analysis should be performed to identify the areas where synergies and efficiencies are possible and then collaborate with stakeholders to create or extend existing mappings between CDISC and other standards, controlled terminologies, and models to represent data originating across different sources.

**Conclusions:**

There are many ongoing data standardization efforts around human health data–related activities, each with different definitions, levels of granularity, and purpose. Among these, CDISC has been successful in standardizing clinical trial-based data for regulation worldwide. However, the complexity of the CDISC standards and the fact that they were developed for different purposes, combined with the lack of awareness and incentives to use a new standard and insufficient training and implementation support, are significant barriers to setting up the use of CDISC standards for RWD. The collection and dissemination of use cases, development of tools and support systems for the RWD community, and collaboration with other standards development organizations are potential steps forward. Using CDISC will help link clinical trial data and RWD and promote innovation in health data science.

## Introduction

### Background

Real-world data (RWD) and real-world evidence (RWE) have an increasingly important role in clinical research and health care decision-making in many countries [1-6]. To leverage RWD and generate reliable RWE, a framework must be in place to ensure that the data are well-defined and structured in a way that is semantically consistent across stakeholders to facilitate learning. The Clinical Data Interchange Standards Consortium (CDISC) RWD Connect Initiative was designed to better understand the barriers to implementing CDISC standards for RWD and to obtain a picture of what tools and guidance may be needed to implement CDISC standards more easily for this purpose.

In the world of traditional clinical trials, which are undertaken with the intent of submitting a new medical product or intervention to regulatory authorities such as the US Food and Drug Administration (FDA) or the Japanese Pharmaceutical and Medical Devices Agency for marketing authorization approval, a set of global data standards has been adopted and is being required by an increasing number of national and regional regulatory agencies. These standards were developed through CDISC, a global nonprofit organization that started >20 years ago to generate open-access platform-agnostic data standards for clinical research and its link to health care.

**Figure 1:**
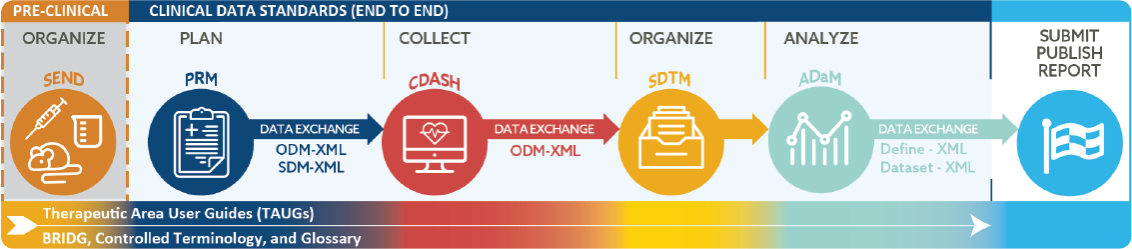
Clinical Data Interchange Standards Consortium standards in the clinical research process. ADaM: Analysis Data Model; BRIDG: Biomedical Research Integrated Domain Group; CDASH: clinical data acquisition standards harmonization; ODM: operational data model; PRM: Protocol Representation Model; SDM: Study Design Model; SDTM: study data tabulation model.

The CDISC standards span the clinical research process and include standards for the exchange of nonclinical data (SEND), data collection case report forms (CRFs; clinical data acquisition standards harmonization [CDASH]), aggregation and tabulation (study data tabulation model [SDTM]), Biomedical Research Integrated Domain Group (BRIDG) logical model, and operational data model (ODM) for transport (Figure 1). In collaboration with the National Cancer Institute’s Enterprise Vocabulary Services (NCI-EVS) program, CDISC has developed a rich controlled terminology that is linked to other common research semantics through the NCI-EVS tools. These standards, presented in data models, implementation guides, and user guides, are globally recognized and heavily used by the biopharmaceutical industry and some academic institutions.

Although there are other standards developed and designed for different purposes (eg, health care data and observational studies), we believe that the benefits of using CDISC standards for purposes outside regulatory submission are many and include improvements in data sharing, cross-study analysis, and meta-analysis of data for all clinical researchers, as well as streamlining the regulatory submission, review, and approval. Please see the Multimedia Appendix 1 of the full RWD Connect report for 4 supportive use cases (Infectious Diseases Data Observatory, Finger Lakes, Pan American Health Organization Hearts, and the Clinical Innovation Network) [7].

Currently, CDISC standards are required for electronic submissions of study data to the US FDA [8] and the Japanese Pharmaceutical and Medical Devices Agency [9] and are recommended by Chinese [10] and European regulators in rare instances where raw data are requested [4]. Government initiatives or centers that fund research also recommend and use CDISC standards, which include the Innovative Medicines Initiative [11], the US National Cancer Institute, and the National Institute of Allergy and Infectious Diseases. In addition, the Japan Agency for Medical Research and Development (AMED) has stated the following:

In the future, clinical trials including investigator-initiated studies will need to comply with the CDISC standards from the planning and implementation stages. Sooner or later, it is expected that we will require the use of CDISC standards for AMED’s contract research

11

Although there are multiple definitions of RWD currently in use, the CDISC glossary has adopted the following:

Data relating to patient health status and the delivery of health care routinely collected from sources other than traditional clinical trials. Examples of sources include data derived from Electronic Health Records (EHRs); medical claims and billing data; data from product and disease registries; biobanks; patient-generated data, including from in-home-use settings; and data gathered from other sources that can inform on health status, such as mobile devices

15

This definition of RWD is similar to the European Medicines Agency (EMA) definition, “routinely collected data relating to a patient's health status or the delivery of health care from a variety of sources other than traditional clinical trials [12].”

**Figure 2:**
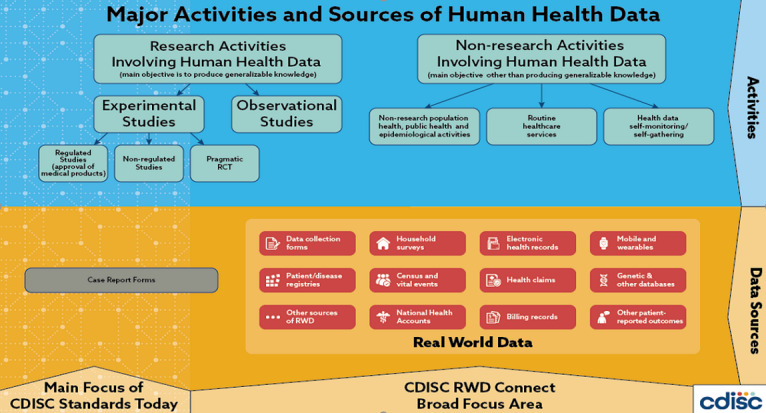
Major activities and sources of human health data. CDISC: Clinical Data Interchange Standards Consortium; RCT: randomized controlled trial; RWD: real-world data.

Figure 2 describes the data sources for RWD as they relate to research and nonresearch activities involving human health data [7]. This diagram was developed in collaboration with the Expert Advisory Board (EAB) members, with a majority consensus, and it is an oversimplification of reality. It would be impractical to attempt to cover all possible sources and types of RWD. Attempts were made to accommodate all suggestions, some of which contradicted each other. The diagram was meant to generate consensus on the main types of data that are considered RWD and their possible data sources. The FDA defines RWE as “the clinical evidence about the usage and potential benefits or risks of a medical product derived from analysis of RWD [1]”. Therefore, if we have a consensus on the definition of RWD, then we believe that the FDA definition of RWE can be applied. Furthermore, we acknowledge that public health activities can involve research activities, which would then be included in the *research activities* on the left of the diagram. Research activities comprise activities using any kind of data, including public health sources and patient registries. The diagram shows that there are some research activities and many nonresearch activities that generate RWD.

There is no single definition of pragmatic randomized controlled trials. Pragmatic design elements exist on a spectrum [13]. Therefore, further discussion on the definition and scope of pragmatic clinical trials is needed to better understand where they fit in the realm of RWD.

Sherman et al [14] proposed the following working definition for RWE, “Information on health care that is derived from multiple sources outside typical clinical research settings, including electronic health records (EHRs), claims and billing data, product and disease registries, and data gathered through personal devices and health applications.” The CDISC Glossary defines RWE as follows, “The clinical evidence derived from analysis of Real-World Data (RWD) regarding the usage and potential benefits or risks of a medical product [15].” The FDA issued a Framework for RWE [3] in December 2018 to announce a program that included demonstration projects, stakeholder engagement, and internal processes to evaluate RWE and promote shared learning and constituency, as well as guidance to assist in using RWD. This framework also states the following, “RWD sources can also be used for data collection and, in certain cases, to develop analysis infrastructure to support many types of study designs to develop RWE, including, but not limited to, randomized trials (eg, large simple trials, pragmatic clinical trials) and observational studies (prospective or retrospective).”

Similarly, the EMA is also exploring ways to leverage RWD in the generation of RWE. In a recent EMA paper, the authors imagined a future that leverages both regulated clinical trials and RWE to assess safety and effectiveness [16].

Insufficient data standardization in academic and public health settings hinders the use of RWD as part of a regulatory submission package. The use of RWD is increasingly being encouraged by regulatory authorities, given the potential of RWD to provide relevant evidence for new drug or product applications. As noted by Califf [17], RWD could complement and enhance the results of clinical trials. The FDA has expressed the need for new research paradigms to break down the barriers between RWD and clinical research so that evidence can be shared rapidly to improve both domains with increased validity and interoperability [18].

Despite their increasing acceptance as part of regulatory submissions, it is commonly acknowledged that RWD are not collected with research as their primary objective. Therefore, there are significant challenges in using and representing these data for research purposes, which can make the analysis of RWD difficult and resource intensive.

There are a number of disparate standards and systems currently in use to support the collection and analysis of RWD. The diverse panoply of common data models (CDMs; eg, Observational Health Data Science–Observational Medical Outcomes Partnership [OMOP], BRIDG, FDA Sentinel, Patient-Centered Clinical Research Network [PCORNet], and Informatics for Integrating Biology and the Bedside (i2b2)), data exchange standards (eg, Health Level 7 [HL7] Fast Health care Interoperability Resources [FHIR], Define-XML and extension CDISC ODM, and SAS V5 XPORT), and terminologies (eg, Systematized Nomenclature of Medicine–Clinical Terms [SNOMED-CT], Logical Observation Identifiers Names and Codes [LOINC], and Current Procedural Terminology coding) in health care settings across electronic health records (EHRs), insurance claims systems, and medical billing systems are all in varying degrees of development and may not be interoperable as they were not designed to meet the requirements of global regulatory submission [19]. A list of collaborations with other standards and initiatives is provided in Multimedia Appendix 2. Meanwhile, in most other academic and public health settings, data are usually collected in a nonstandard way using different formats and different terminologies [20], which do not allow for the data to be consolidated, compared, and shared. In cases where data are standardized, the variety of approaches, including openEHR, the US National PCORNet, Informatics for Integrating Biology and the Bedside (i2b2), OMOP, and HL7 FHIR, can lead to standard-specific silos. This disconnect creates an evidence gap that slows scientific and public health advances [21]. The need to coordinate across standards is clear, and organizations such as the ISO Joint Initiative Council provide forums to coordinate across standards development organizations; however, these need more support, participation, and adoption.

The benefits of the implementation of standards for RWD are potentially many and include better documentation of data collection, enabled analysis processes, and data sharing [22]. In response, multiple initiatives and tools have been developed in the last few years to seize the opportunity and tackle the challenges resulting from the sudden accessibility of massive amounts of information from multiple RWD sources. For example, in rare diseases where there are many small data collection efforts underway but large regulated clinical trials may not be feasible because of insufficient patient numbers and ethical issues, being able to combine or compare data from different sources becomes even more critical [23]. Cancer is another therapeutic area where there are efforts underway to pool and share data. The National Cancer Institute Cancer Research Data Commons (CRDC) is an infrastructure that connects data sets with analytics tools to allow users to share, integrate, analyze, and visualize cancer research data to drive scientific discovery.

### Objective

With these potential benefits in mind and considering the increasing need and interest in data standardization beyond regulatory submissions, CDISC created the CDISC RWD Connect Initiative to develop a vision and strategy for the implementation of CDISC standards for RWD [7]. The first step of the CDISC RWD Connect Initiative was to invite international experts to join an EAB and to involve them in the Delphi survey process described in this paper to better understand what it will take to achieve CDISC standards implementation beyond regulatory submissions.

## Methods

### Overview

The goal of the RWD Connect initiative was to listen to the stakeholder community to better understand the barriers to implementing CDISC standards for RWD and to get a picture of what tools and guidance may be needed to more easily implement CDISC standards. The second phase focused on creating a strategy for fostering the consistent implementation of CDISC standards within the academic community. In addition, the initiative identified concrete examples of the use of CDISC standards for RWD and worked collaboratively with the implementers to document the use cases, their scope and characteristics, challenges, and lessons learned. With these goals in mind, we chose to conduct a qualitative Delphi survey to collect an array of different opinions about the use of CDISC standards for RWD and to assess the level of agreement or disagreement on key issues in an asynchronous, global manner. The results from the Delphi and the use cases were the foundation for the proposed vision and strategy described in this manuscript [7].

### Qualitative Delphi

In September 2019, the CDISC RWD Connect formed an EAB with key stakeholders. The criteria were knowledge of CDISC standards (any level) and experience working with RWD. In selecting candidates, an effort was made to balance the different regions of the world to the extent possible and to include experts from academia, government, regulators, and health care settings. A list of EAB members is provided in Multimedia Appendix 3.

**Figure 3:**
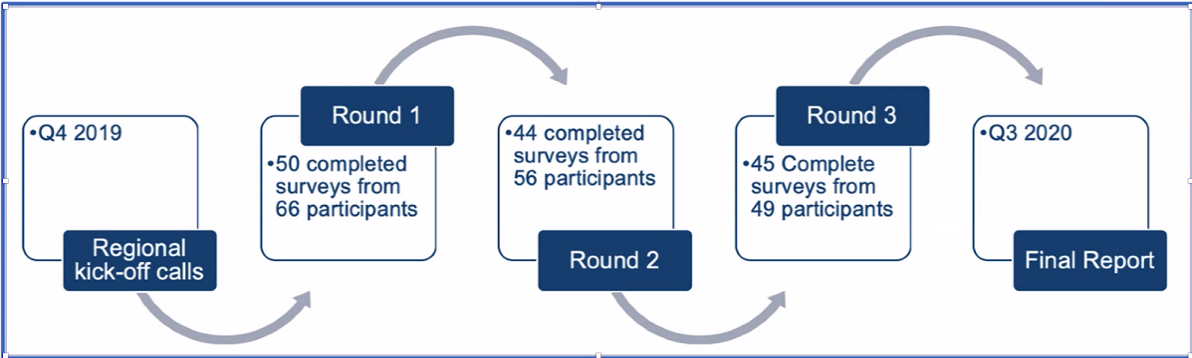
Rounds and participants of the modified qualitative Delphi process.

We identified an initial list of potential members who were either already CDISC partners or collaborators or had been referred by a partner or collaborator. We sent out email invitations to these 70 individuals inviting them to join the initiative, with a required commitment to participate in 3 qualitative Delphi rounds and a final web-based to discuss the results and agree on a way forward. Of the 77 experts invited to participate, 66 (86%) participated in round 1, 56 (73%) participated in round 2, and 49 (70%) participated in round 3 (Figure 3). All EAB members were invited to join the writing committee, and those who accepted are the coauthors of this paper.

From October 2019 to May 2020, we conducted a modified version of a 3-round qualitative Delphi survey based on published methodology [24]. The goal of the CDISC RWD Connect modified qualitative Delphi survey process was to answer the following questions: what are the priorities, needs, and challenges around the use of CDISC data standards outside regulated clinical trials; how can CDISC minimize the barriers to implementing CDISC standards for RWD; and what are the requirements for potential tools and educational materials for implementation support? The Delphi questionnaire is presented in Multimedia Appendix 4.

In November 2019, the first round of the qualitative Delphi survey was sent to the EAB. The survey comprised 2 sections: section 1 with questions for background information and section 2 with questions for the generation of group statements, as described in the CDISC RWD Connect: Report of Qualitative Delphi Survey [7]. During this first round, we received 50 answered surveys, which included perspectives and insights from at least 66 participants globally (at least 8 answered surveys had consolidated answers from multiple people within a team). From the responses obtained from the first round of the qualitative Delphi, we developed a summary of *group statements* containing the prevailing views of the EAB.

In February 2020, a second round of the qualitative Delphi survey was sent to the EAB. In it, participants were provided with group statements and were given a chance to state whether they agreed with each group statement and how they would modify it. We did not add any new questions. During the second round, we received 44 completed surveys from 56 participants.

In April 2020, the third and final round of the qualitative Delphi survey was sent to the EAB, and participants had a chance to review the final version of the group statements and share whether they strongly agreed, moderately agreed, or disagreed with each of the statements and their reasons for the same. During the final round, we received 45 completed surveys from 49 participants.

### Use Cases

Examples are an effective way of showing how CDISC standards can be deployed in use cases outside regulated clinical trials. There are creative and innovative studies already being performed globally in various organizations that make use of CDISC standards. A key part of this study was to collect a number of these use cases from CDISC’s existing network of partners and collaborators, as well as to ask for use case recommendations from the EAB.

To collect information on the selected use cases, we performed phone interviews and reviewed databases, presentations, and other documentation relevant to the experience of using CDISC standards for RWD [7].

## Results

### Overview

In total, 66 experts were included, and 139 feedback instances were collected and analyzed. There was broad agreement that the standardization of RWD is necessary, and the primary focus should be on its ability to improve data sharing and the quality of evidence. The RWD diagram shown in Figure 2 was extensively discussed by the EAB through the Delphi process. Approximately 49% (32/66) of the participants strongly agreed with the final version of the diagram, 42% (28/66) moderately agreed, and 9% (6/66) disagreed.

The priorities of data resources for the CDISC RWD Connect Initiative, as agreed strongly among the experts, included EHRs with a particular interest in data shared using the HL7 FHIR standard, data stemming from observational studies, and wearable devices and patient-reported data. The experts recommended that a gap analysis be performed, as there are different standardization stakeholders in these areas. An official mapping between CDISC and other standard terminologies and a common model to represent the data across different sources was considered necessary. Efforts have been undertaken to fill this void, such as the BRIDG model work group and the FDA CDM Harmonization project [25,26]. This work could be extended to use CDISC as a common model based on existing standards. The duplication of effort should be avoided where possible.

### Participants’ Background Information

During the first round of the qualitative Delphi survey, which was the most comprehensive and had the greatest impact on the results of this process, we received 50 answered surveys, which included the perspectives and insights from at least 66 participants globally (at least 8 answered surveys had consolidated answers from multiple people within a team). The respondents represented the following continents: Americas, 49% (32/66); Asia, 29% (19/66); Europe, 20% (13/66); and Africa, 2% (1/66). Regarding the represented institutions, 34% (22/66) of the participants represented universities, 24% (16/66) government organizations, 15% (10/66) research centers, 13% (9/66) nonprofit organizations, 6% (4/66) international organizations, and 8% (5/66) others, including hospitals, software companies, and other enterprises. Approximately 95% (63/66) of the participants had experience with RWD, with varying degrees of expertise (Table 1). The *Acknowledgments* section contains a list of institutions represented in the EAB.

**Table 1:** Expert advisory board participants’ experience with real-world data (RWD; not mutually exclusive; N=66).

Participant experience	Participant, n (%)
I have conducted experimental research or academic studies using RWD that were not intended for regulatory submission.	27 (21)
I have conducted observational research studies (cohort study and case control etc).	24 (19)
I have worked with routine health care data.	24 (19)
I have worked with public health data (surveillance and public health programs).	20 (16)
I have worked with multiple RWD sources to conduct research around health care delivery.	17 (13)
I have not worked with RWD.	6 (5)
I attempted to use RWD data but gave up because of challenges.	1 (1)
Other	9 (7)

### Benefits and Opportunities From Standardization of RWD

We also asked what participants saw as the primary benefits and opportunities from the standardization of RWD, and specifically, how they would make this case to their colleagues. Most participants (53/66, 80%) strongly agreed that the primary benefits and opportunities from RWD standardization focused on the ability to share data and improve the data quality. Specifically, they stressed that a CDM with no additional mapping was important. As one respondent stated, “achieving accurate results requires a common language, harmonization, and codified and structured data.” Respondents acknowledged that implementing standards requires significant investment. However, the use of data standards and vocabularies could enable standard data collection, machine readability, automated data extraction from EHRs, data pooling, an increase in statistical power and scalability (especially for neglected or rare diseases), reproducibility, and allow long-term follow-up of a clinical trial. All of these benefits could be achieved while saving time and effort, enhancing productivity, and speeding the publication of results, which essentially enables findability, accessibility, interoperability, and reusability (FAIR) data principles [27,28].

Participants also noted that with the increased standardization of RWD, there might be an opportunity to better understand RWD and to improve or optimize the study design, which could facilitate more research studies being able to use RWE to support regulatory decision-making. Participants also noted that standards would be key to using data acquired via devices, especially in *Bring Your Own Device* research, and for leveraging other sources of data (eg, claims data). Standards can also increase consistency in clinical trial initiation and execution in both academic and industry settings, which could speed the development of new therapies and treatments. Others noted that standards could reduce the cost of archiving and long-term storage of data, allow for ethics and privacy protection to be more strictly addressed, and contribute to the learning health care system [29,30].

### Priority Components for CDISC RWD Connect

We asked participants to share which types of RWD CDISC should focus on first and why. Below is a summary of the participants’ answers after 2 rounds of revisions based on the feedback received. Of note, 62% (41/66) of the participants strongly agreed with the following summary of the priorities and the rationale, and 38% (25/66) moderately agreed with them.

The responses to why CDISC should prioritize EHRs were as follows: EHRs are one of the most available and largest data sources; EHRs are already in electronic format; EHRs contain important and essential information directly relevant to the patients’ health status; it would allow us to identify how EHRs could be improved to support better RWE; and EHRs will be the hardest to implement but the most important source of data for the generation of RWE.

The responses to how CDISC should prioritize the harmonization of their standards with HL7 FHIR were as follows: by harmonizing CDASH data elements with FHIR; by working with HL7 working groups to connect clinical research with health care, to update FHIR resources, or develop new FHIR resources needed for research; and by creating a canonical representation of FHIR in CDISC ODM as the electronic data capture vendors will likely be using ODM to ingest and share data from EHRs.

The responses for why CDISC should prioritize observational study data were as follows: observational study data are far less developed in terms of standard use compared with EHRs, and observational studies and pragmatic clinical trials collect similar data to randomized clinical trial data that can be leveraged to inform clinical or policy decision making [31]; CDISC should collaborate with Observational Health Data Science and Informatics (OHDSI)–OMOP CDM on observational research data; as standardized data can be shared and reused more broadly, observational studies using standards will have a greater impact; and the OMOP CDM is a standard-on-the-rise (for observational studies) that should be considered.

Secondary areas of focus should include data commons, registries, mobile health (including automatically generated data), billing records, and medical claims data.

The EAB also mentioned that before broadening the scope of CDISC, a gap analysis and insight into other standardization stakeholders should be conducted. There are already many standards for some of the areas above and often institutional standards as well. At a minimum, to help aggregate and analyze data from these different systems, a published mapping between CDISC controlled terminology and other standard terminologies used for the same data element might be useful; however, the potential lack of equivalence could be problematic. Given that many standard terminologies used in health care do not contain explicit definitions for the concepts contained therein, these mappings could potentially improve those terminologies as CDISC defines all of its controlled concepts. CDISC should also focus on the fundamentals of how to model and represent data and how to manage changes. Unless these are done well, building new additional standards on top of poor foundations will not necessarily bring any benefit. It was the opinion of some on the EAB that some CDISC models have underlying principles, mainly in the areas of data types and data modeling, that can make the implementation challenging. The EAB has recommended augmenting and extending CDISC standards with generalized forms and classes of RWD to address these issues.

### Standards for Devices and Wearables

There are significant challenges related to implementing data standards for innovative data collection technologies, such as consumer wearables (eg, Fitbit [Google LLC], Apple Watch [Apple Incorporated], and other monitoring devices). The data itself suffers from credibility, accuracy, and reliability issues associated with proprietary, nonclinically tested algorithms that differ across vendors. This naturally leads to interoperability issues when comparing the same data across different devices; that is, given two different proprietary algorithms, one cannot say that a heart rate measurement is the same across two different devices. Concern was also expressed around privacy, data ownership, and inequitable access, which may leave certain populations out of the analysis. Finally, the current direct-to-consumer marketing approach ensures that there is very little incentive for competing companies to standardize and harmonize among each other.

### Patient Perspectives in RWD

There was general agreement that the perspective of the patient, with respect to the collection and use of RWD, is vitally important to ensure the ethical use of the data. However, there was no consensus as to whether the patients' perspective regarding the use of data standards was relevant. At the very least, it was thought that data standards should enable data sharing with the patients themselves and help clinicians make decisions about patient care. Collaboration with professional organizations such as clinical medical societies and disease foundations, as well as patient advocacy groups, was thought to be of value in this effort. A good place to start with respect to patient-valued data standardization was with the standardization of patient-reported outcome data models and measures. Another potential resource currently under development is the Critical Path Institute’s *Best Practice Recommendations for ePRO Dataset Structure and Standardization to Support Drug Development*, which uses CDISC standards.

In addition, patient groups should be educated about the benefits of data standards and how this can lead to better and more efficient data sharing. Increasing patients’ awareness of the usefulness of the data for themselves and for knowledge generation would ensure strong, patient-lead advocacy groups that promote data standards.

### Making the Case for Using CDISC Standards for RWD

Participants were asked what they saw as the main challenges in academic clinical research that could be overcome with the increased standardization of RWD. Their responses focused on issues related to the different sources of data, inconsistency in data collection, inconsistencies in the data, text fields, poor integration and interoperability, too many standards used or none at all, no standards analysis or meta-analysis tools that results in the development of in-house standards, mapping and the accompanying loss of data or errors, and finally, lack of awareness regarding standards and harmonization of clinical trial initiation and conduct across academic clinical research sites, all of which contribute to the creation of data silos.

### Tools or Support Needed

We asked participants what tools or support would be helpful in the implementation of CDISC standards to support RWD in academic settings. The responses focused on providing data collection templates, CDMs, standard user guides, and dictionaries. It was reported that data collection templates containing preannotated fields that link data collection activities to CDISC standards would be useful. In addition, CDISC standards would need to be expanded to include those elements commonly collected and analyzed in observational studies. Finally, educational and training opportunities for CDISC standards will be required to support those working in academic research.

Robust software tooling would also be needed to enable efficient data collection, mapping, quality control or validation, integration, transformation, and analysis. Ideally, software tooling should be open-source, easy to use, flexible, and web-based, containing CRFs and ODM files with built-in CDASH and SDTM coding. Given the heterogeneity of systems used across health care and academic institutions, novel software tooling should be able to interact with the existing standards, such as HL7 FHIR. Mapping across data elements and dictionaries to marry in-house standards with CDISC-standard variables and terminology would also be a useful feature in any software tool. Terminology and metadata validation tools based on open-source Export, Transform, and Load (ETL) tooling may help with quality control issues. These tools would also need to be usable and supported by regulatory agencies.

### Building Knowledge and Expertise on CDISC Implementation

We also asked about the most effective ways to build knowledge and expertise on the implementation of CDISC standards in academic institutions. The responses included providing funding for capacity building (eg, award grants to academic institutions and fund institutional roles to support implementation). One respondent noted that CDISC has a role in communicating with research investors or funders to streamline requirements and competing standards across funder organizations. Other recommendations included collaboration and compromise with and among institutions, creation of a certification program, development of simple, free web-based tools (eg, templates for CRFs, data dictionaries, and data sets based on real-world scenarios), documenting and highlighting the use cases and demonstrations, and providing web-based and on-site training.

## Discussion

### Principal Findings

Existing standards support many facets of human health activity–related data and clinical research. However, there is a lack of standardization for the process to derive RWE from RWD, which results in limited use of RWD in clinical medicine and therapeutics development. CDISC standards have been successfully used in trial-based data management for regulated research worldwide. CDISC aims to extend its standards to support RWD to bridge the existing gaps. However, the complexity of CDISC standards, lack of awareness and incentive to use a new standard, and insufficient training and implementation support were reported to be barriers to setting up standards for RWD following the CDISC methodology, although CDISC has been successful in the trial-based data area. As commented, potential solutions include building use cases for using CDISC for RWE studies, developing tools and support systems, and collaborating with other standards and initiatives (Multimedia Appendix 2).

### Barriers to the Use of CDISC Standards for RWD

EAB participants identified the most significant barriers to using CDISC standards in academic settings for RWD. First, it was reported that CDISC standards were considered to be more complex than those used currently for RWD and that their implementation in an academic setting might be burdensome because of unstructured data. There are likely insufficient financial and trained human resources within academic institutions to put toward an implementation. Granting agencies should consider including resource allocation for the use of data standards within their awards. Free, open-source, and easy-to-use tools that incorporate CDISC standards, as well as free or reduced-price training, could also be used to support the implementation of data standards within academic institutions.

Second, there are real gaps in CDISC standards related to RWD that prevent their use in fully supporting RWD at this time. It was the opinion of some on the EAB that some CDISC models have underlying principles, mainly in the areas of data types and data modeling, that can make their implementation challenging. For example, data elements related to longitudinal, prospective, and observational study designs are not sufficiently modeled in CDISC standards currently. The EAB recommended augmenting and extending CDISC standards with generalized forms and classes of RWD to address these issues. A gap analysis between CDISC and OMOP data elements could be the first step in reducing the disparity.

Third, there may be insufficient knowledge of the value of data standards, and more specifically, CDISC standards, coupled with a lack of real and perceived incentives for using standards within institutions such that implementation of CDISC standards may be considered a low priority. In addition, the value of the use of CDISC standards, which has been established in certain sectors (eg, pharmaceutical industry), might not be as well known outside of the regulated research context. An increase in public presentation and publication of case studies showing the enormous value of CDISC standards would go a long way toward educating groups outside of the pharmaceutical industry.

Finally, RWD is currently supported by a number of disparate CDMs, standards, and terminology in use by EHRs, insurance claims systems, and medical billing systems in varying degrees of development; however, they are not connected to one another. These data models, standards, and terminologies are not usually the same as CDISC, which would require harmonization or mapping to remedy. Currently, there is little incentive for EHR vendors and health care providers to adopt data standards. Furthermore, academic institutions may lack CDISC-trained human resources, which would require financial and temporal resources to remedy. In addition, academic institutions may use multiple disparate systems within and across organizations that would disallow standardization even within a single institution. There is also insufficient knowledge on the importance of data standards and, more specifically, a perceived lack of benefit to using CDISC standards beyond reporting to regulatory agencies. For example, journal publication of results does not require the use of data standards of any kind.

### The Future of RWD and CDISC Standards

CDISC initiated the CDISC RWD Connect Initiative with the aim of developing a vision and strategy for the use of CDISC standards for RWD. The following is a list of the key requirements and steps to achieve this goal:

- Simple and flexible tools (eg, templates, plug-and-play tooling, master user guide for mapping and terminology, and open-source file formats)
- Free or affordable training and education (eg, quick start for academics, one-to-one training to create new resources or apps, or registries using CDISC standards)
- Support for standardization of EHR data (eg, decrease the use of open text fields in EHRs to facilitate artificial intelligence data extraction from physician’s notes, use new terminologies, and collaborate with health care standards experts and vendors to align and design systems that bridge the health care to research gap) while being mindful of the fact that the primary role of EHRs is patient care, and this process should, therefore, minimize the impact on providing that care
- Publication of use cases that demonstrate the value in the use of CDISC standards outside regulated clinical trials
- Standardization across terminologies used by health care and research
- Simplification where possible and minimizing the number of standards
- Regulation and requirements; specifically, where data cannot be standardized at collection, regulatory requirements must be established to confirm the validity of the mapped data
- Ongoing support for implementation (eg, information technology staffing, 24-hour support, data standards experts, and data warehouse expertise in staff to help implementers)
- Champions and key opinion leaders to support or influence the use of standards and cooperation
- Development of a well-defined purpose and scope for the use of CDISC standards for RWD
- Financial support for development, maintenance, and implementation; specifically, resources are needed for implementation support in the form of educational programs and consulting services
- Incentives in the form of grants to consortiums implementing CDISC standards, free education, free CDISC membership, and granters allowing budget lines for the use of standards and other funding mechanisms can also help encourage the use of CDISC standards

### Areas of Nonagreement During the Qualitative Delphi Process

For the most part, EAB participants were able to reach a consensus on the main areas of discussion. However, there were some specific issues on which consensus was not reached. Participants had different ideas regarding the types and sources of data that could be considered RWD. Most participants agreed that RWD standardization efforts should focus on EHRs as a priority. However, the few who strongly disagreed explained that the implementation of CDISC standards in EHRs would be difficult and that HL7 FHIR was addressing the EHR space. Registries were another area of nonagreement, with some participants prioritizing registry data standardization and others saying it should not be a priority. Finally, some participants maintained that CDISC standards should be made easier to use before attempting to expand their scope, whereas others proposed improving the standards in parallel with exploring and testing the expansion of use for RWD.

### Limitations

This survey was sampled by convenience; therefore, we were not able to generalize the results of the survey to all settings of RWD generation and use. This project was also geographically limited, as most participants originated from North America and Europe and to a lesser extent from Asia and Africa. We note that the risk for bias is present because of the reasons for which people chose to take part in the Delphi survey.

### Conclusions

The CDISC RWD Connect project sought to better understand the barriers to implementing CDISC standards for RWD and to articulate steps toward making CDISC standards easier to use in settings outside regulated clinical trials. Recommendations included identifying the tools and guidance needed for consistent implementation and the expansion of CDISC standards to accommodate data stemming from observational studies, which account for a large amount of available clinical data. Other potential standards development focus areas included data commons, registries, mobile health, and billing and medical claims.

Other practical steps included bringing the standards up to date with current data science technology, making implementation guides easier and more intuitive to be implemented by novice users, and creating a number of tools, strategies, and adaptations to facilitate and promote the use of RWD. Examples included augmenting the SDTM with generalized forms and classes of RWD, creating simpler and more flexible templates and tools, providing free or affordable training and education, increasing regulations and requirements for RWD standards, encouraging champions and financial support, and disseminating concrete examples of the implementation of CDISC standards for RWD. Underpinning these steps, CDISC should support a community of practice that highlights successful implementations and shares their experience by publishing use cases and presenting at conferences and connectathons. Finally, global regulatory support and mandates from funders of academic studies were also cited as key factors in fostering implementation.

There is a unique opportunity for CDISC to broaden the scope of its suite of data standards to accommodate and connect with RWD to better facilitate RWD sharing. We believe that CDISC standards can provide FAIR structure and semantics for common clinical concepts and domains and help bridge the gap between RWD and clinical trial–generated data for the benefit of all stakeholders. CDISC will use the findings and recommendations from the RWD Connect initiative as inputs to their strategic plan and take the next steps toward developing standards, tools, and guidance for the use of RWD in global regulatory submissions.

## Appendix

Multimedia Appendix 1 Clinical Data Interchange Standards Consortium Real-world Data Connect Report of Final Qualitative Delphi Survey Consultation to Expert Advisory Board.

Multimedia Appendix 2 Collaborations with other standards and initiatives.

Multimedia Appendix 3 List of expert advisory board members.

Multimedia Appendix 4 Delphi questionnaire.

## Abbreviations

AMED: Japan Agency for Medical Research and Development
BRIDG: Biomedical Research Integrated Domain Group
CDASH: clinical data acquisition standards harmonization
CDISC: Clinical Data Interchange Standards Consortium
CDM: common data model
CRDC: Cancer Research Data Commons
CRF: case report form
EAB: expert advisory board
EHR: electronic health record
EMA: European Medicines Agency
ETL: Export, Transform, and Load
FAIR: findability, accessibility, interoperability, and reusability
FDA: Food and Drug Administration
FHIR: Fast Health care Interoperability Resources
HL7: Health Level 7
i2b2: Informatics for Integrating Biology and the Bedside
LOINC: Logical Observation Identifiers Names and Codes
NCI-EVS: National Cancer Institute’s Enterprise Vocabulary Services
ODM: operational data model
OHDSI: Observational Health Data Science and Informatics
OMOP: Observational Medical Outcomes Partnership
PCORNet: Patient-Centered Clinical Research Network
RWD: real-world data
RWE: real-world evidence
SDTM: study data tabulation model
SEND: standards for the exchange of nonclinical data
SNOMED-CT: Systematized Nomenclature of Medicine–Clinical Terms
