# Supplementary material for: Use of Clinical Data Interchange Standards Consortium (CDISC) Standards for Real-world Data: Expert Perspectives From a Qualitative Delphi Survey
Source: JMIR Med Inform. 2022 Jan 27;10(1):e30363. doi: 10.2196/30363 (PMC8832264; doi:10.2196/30363)
Supplement: Multimedia Appendix 1 [file medinform_v10i1e30363_app1.docx]

**
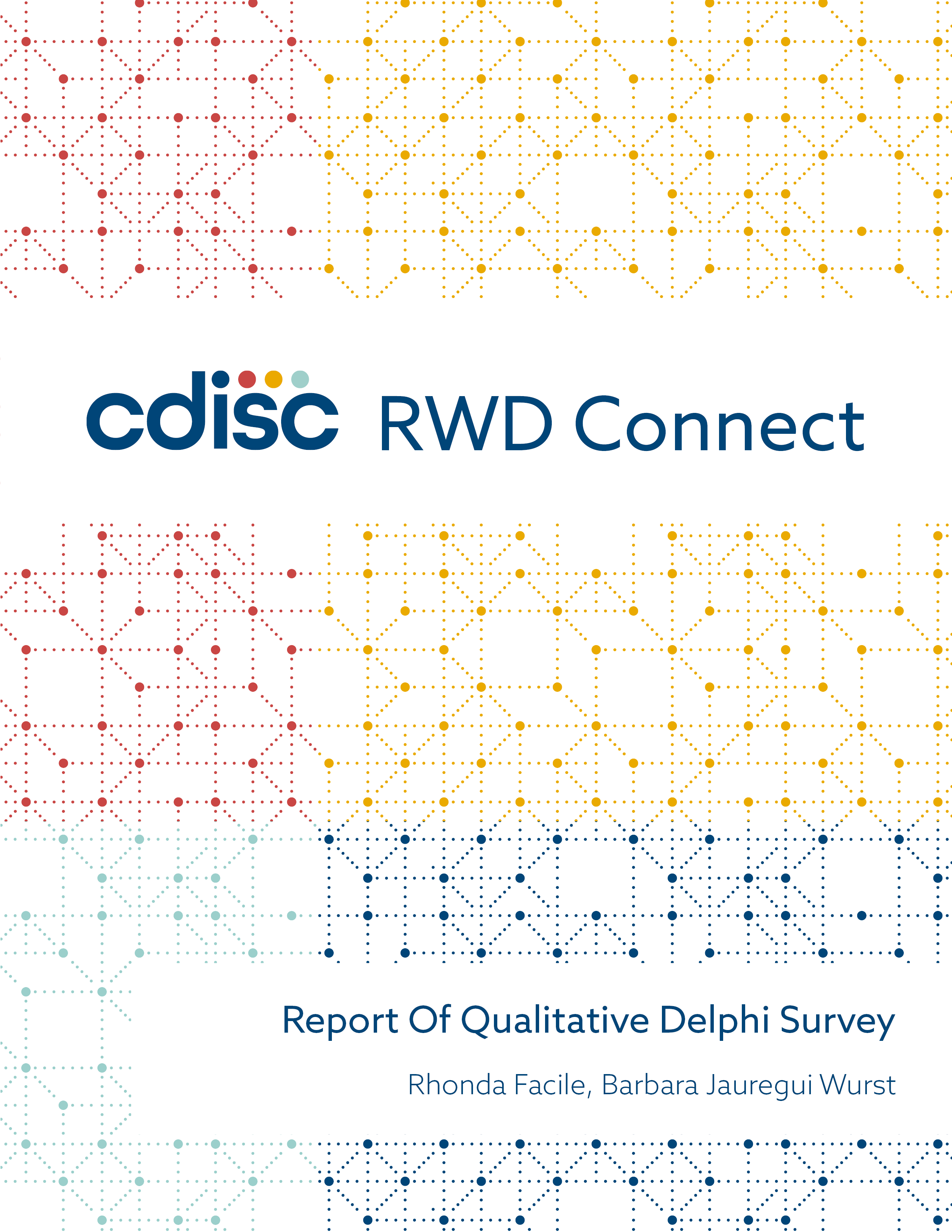
**

**CDISC RWD Connect Report of Qualitative Delphi Survey Consultation to Expert Advisory Board**

Table of Contents

[Executive summary 4](#_Toc70425721)

[Introduction 5](#_Toc70425722)

[Participants Background Information 6](#_Toc70425723)

[Participant Experience with Real-World Data (RWD) 8](#_Toc70425724)

[Participants Experience with Data Standards 8](#_Toc70425725)

[Survey Results 11](#_Toc70425726)

[CDISC Focus and RWD 11](#_Toc70425727)

[Priority Components for CDISC RWD Connect 11](#_Toc70425728)

[Barriers to the use of CDISC Standards for RWD 14](#_Toc70425729)

[Steps Needed for Implementation of CDISC in Academia 16](#_Toc70425730)

[Making the Case for Using CDISC Standards for RWD 18](#_Toc70425731)

[Tools or Support Needed 19](#_Toc70425732)

[Benefits and Opportunities from Standardization of RWD 21](#_Toc70425733)

[How to Build Knowledge and Expertise on CDISC Implementation 24](#_Toc70425734)

[How to Reward and Promote the Use of CDISC Standards in Academia 25](#_Toc70425735)

[Standards for Devices and Wearables 27](#_Toc70425736)

[Patient's Perspective in RWD 29](#_Toc70425737)

[Collaborations with Other Standards and Initiatives 31](#_Toc70425738)

[The Future of RWD and CDISC Standards 34](#_Toc70425739)

[Use Cases 41](#_Toc70425740)

[IDDO Use case – Novel SDTM implementation to Improve Legacy Data Sharing 41](#_Toc70425741)

[Finger Lakes Use Case - Application of SDTM to the NY State Perinatal Data System 44](#_Toc70425742)

[PAHO HEARTS Use Case – Mapping of Hypertension Program Data into CDASH 46](#_Toc70425743)

[Clinical Innovation Network (CIN) Use Case – Gap Analysis between Remudy and TAUG-DMD (and related the other CDISC Standards) to Construct Patient Registry for International Data Sharing 48](#_Toc70425744)

[References 52](#_Toc70425745)

[Appendix A – List of EAB Members 53](#_Toc70425746)

[Appendix B – Survey Questions 55](#_Toc70425747)

**CDISC RWD Connect Report of Qualitative Delphi Survey Consultation to Expert Advisory Board**

Authors: Rhonda Facile, M.S.^1^, Barbara Jauregui Wurst, M.D., M.S.^2^

^1^ Vice-President, Partnerships and Development, CDISC

^2^ Academic and Public Health Advisor (consultant), CDISC

# Executive summary

The CDISC RWD Connect project was initiated in November 2019, with the goal of engaging the academic community, and the broader CDISC global community, to better understand the barriers to implementing CDISC standards for Real World Data (RWD) and to get a picture of what tools and guidance may be needed to more easily implement CDISC standards. A modified qualitative Delphi Survey methodology was chosen to facilitate asynchronous global data collection. The final phase of this effort will be to incorporate the recommendations into the CDISC strategic plan and develop the necessary tools and guidance.

The survey was conducted in two rounds: Round 1 consisted of questions about background information (e.g. relevant institutions represented, experience with RWD, standards, and CDISC standards, etc.), round 2 consisted of questions for the generation of group statements. During the first round, 50 answered surveys were received, which included the perspectives and insights from 66 participants globally (eight answered surveys comprised consolidated answers from multiple people within a team). We developed a summary of “group statements”, containing the prevailing views of the Expert Advisory Board (EAB) from the responses obtained from the first round of the qualitative Delphi.

The EAB was highly responsive; we received rich and thoughtful responses that are detailed in this report. The recommended priority areas identified via the qualitative Delphi were that CDISC should focus on connecting with Electronic Health Records (EHRs), with Health Level 7- Fast Healthcare Interoperability Resources (HL7-FHIR) and with data stemming from observational studies. Other focus areas identified included data commons, registries, mobile health, and billing and medical claims data (US). The EAB also recommended that a number of tools, strategies and adaptions be created to facilitate the use of RWD. Examples include augmenting SDTM with generalized forms and classes for RWD, simpler and more flexible templates and tools, free or affordable training and education, increasing regulation and requirements for standards for RWD, champions and financial support, and developing use cases to showcase the implementation of CDISC standards for RWD.

During the consultation process, a few use cases were collected via phone interviews to illustrate the implementation of CDISC standards outside regulated clinical trials. CDISC intends to continue to collect these use cases and make them available to the CDISC community.

# Introduction

In 2018, a CDISC Blue Ribbon Commission was created to shape and guide CDISC’s vision and strategy. Composed of global leaders from academia, the pharmaceutical industry, government agencies (including regulatory bodies), patient foundations and fellow standards development organizations, the Blue Ribbon Commission developed a list of recommendations to support CDISC ‘s suite of clinical data standards now and in the coming decade. One of the key recommendations from the Blue Ribbon Commission was to involve the academic community in a conversation around how CDISC standards can be effectively and efficiently deployed in Real World Data (RWD) settings. The benefits of connecting RWD to CDISC standards are myriad and include improvements in data sharing, cross-study analysis, and meta-analysis of data for all clinical researchers.

Accordingly, CDISC initiated the “CDISC RWD Connect” project to engage the academic community. The first phase of this initiative was to listen to the academic community to better understand the barriers to implementing CDISC standards for RWD and to get a picture of what tools and guidance may be needed to more easily implement CDISC standards. The second phase focused on creating a strategy for fostering consistent implementation of CDISC standards within the academic community. The final phase of this effort will be to incorporate the recommendations into the CDISC strategic plan and make the tools and guidance that are needed a reality.

With these goals in focus, the CDISC RWD Connect team carried out a modified qualitative Delphi survey process with key stakeholders who formed the CDISC RWD Connect Expert Advisory Board (EAB). In November 2019, the first round of the qualitative Delphi survey was sent to the EAB. The survey was conducted in two rounds: Round 1 with questions for background information and round 2 with questions for the generation of group statements. During this first round, we received 50 answered surveys, which included the perspectives and insights from 66 participants globally (at eight answered surveys comprised consolidated answers from multiple people within a team). We developed a summary of “group statements”, containing the prevailing views of the EAB from the responses obtained from the first round of the qualitative Delphi.

In February 2020, a second round of the qualitative Delphi survey was sent to the EAB. In it, participants were provided the group statements and were given a chance to state whether they agreed or not with each group statement, and how they would modify it. During this second round we received 44 completed surveys from 56 participants.

In April 2020, the third and final round of the qualitative Delphi survey was sent to the EAB, and participants had a chance to review the final version of group statements and share whether they strongly agreed, moderately agreed, or disagreed with each of the statements and why. During this final round we received 45 completed surveys from 49 participants.

The present report is a consolidation of the results of the qualitative Delphi consultation, describing the EAB’s views and recommendations for a way forward with CDISC standards and the world of RWD.

# Participants Background Information

**Regions Represented**

The first round of the qualitative Delphi survey was the most comprehensive in terms of data collection and therefore had the greatest impact on the results presented in this report. We received 50 completed surveys, which included the perspectives and insights from 66 participants globally (eight completed surveys contributed consolidated answers from multiple people within a team). Respondents represented the following continents:

- Americas - 49%
- Asia - 29%
- Europe - 20%
- Africa - 2%

**Institutions Represented**

| University | 24 | 34% |
| --- | --- | --- |
| Government organization | 17 | 24% |
| Research center | 11 | 15% |
| Non-profit organization | 9 | 13% |
| International organization | 4 | 6% |
| Other... | 6 | 8% |
| Funder | 3 |  |
| Hospital | 1 |  |
| Software company | 1 |  |
| Enterprise | 1 |  |

| **Region** | **Academic, Research and Other Organizations** | | **Governmental Institutions** |
| --- | --- | --- | --- |
| Americas | Harvard Medical School  University of California at San Francisco  Duke University  University of Chicago  University of Arkansas  George Washington University  University of Iowa  University of California, Berkeley | Pan American Health Organization / World Health Organization  Patient-Centered Outcomes Research Institute (PCORI)  Massachusetts General Hospital  Cedars-Sinai Medical Center  SNOMED  HL7  SAS Institute  C-Path | US Centers for Disease Control (CDC)  Food and Drug Administration – FDA CDER  Food and Drug Administration – FDA CBER  National Cancer Institute (NCI)  National Institute of Neurological Disorders and Stroke (NINDS)  National Institute of Allergy and Infectious Diseases (NIAID)  National Institutes of Health- Enterprise Vocabulary Services (NIH-EVS) |
| Europe | Oxford University  University of Liverpool  University of Newcastle  University of Essex  University of Amsterdam, Netherlands | Innovative Medicines Initiative (IMI)  Liverpool Clinical Trials Research Centre  UK Data Archive  Nestle  Dannon | European Medicines Agency (EMA) |
| Asia | Daegu Catholic University Medical Center, South Korea  NHO Nagoya Medical Center  Sapporo Medical University  University of Tokyo  Institute of Health Data Infrastructure for All (IDIAL)  National Center of Neurology and Psychiatry, Japan  National Cancer Center Hospital East  Japan ARO Council | Xiyuan Hospital, China  Academy of Chinese Medical Sciences  National Rare Disease Registry System of China  Digital China Health Technologies  Mahidol Oxford Tropical Medicine Research Unit (MORU), Thailand  Peking University Clinical Research Institute  Peking Union Medical College Hospital  LinkDoc Technology, Beijing | Pharmaceutical and Medical Devices Agency (PMDA)  Japan Agency for Medical Research and development (AMED)  National Institute of Public Health (Japan) |
| Africa | KEMRI – Wellcome Trust Kenya | | |
| Funding Organizations | The Leona M. and Harry B. Helmsley Charitable Foundation (US)  Bill and Melinda Gates Foundation (US)  Wellcome Trust (UK)  Cohen Veterans Network (US) | | |

## Participant Experience with Real-World Data (RWD)

| I have conducted experimental research/academic studies using RWD that were not intended for regulatory submission. | 27 | 21% |
| --- | --- | --- |
| I have conducted observational research studies (cohort study, case-control, etc.). | 24 | 19% |
| I have worked with routine healthcare data. | 24 | 19% |
| I have worked with public health data (surveillance, public health programs, etc.). | 20 | 16% |
| I have worked with multiple RWD sources to conduct research around healthcare delivery. | 17 | 13% |
| I have not worked with RWD | 6 | 5% |
| I attempted to use RWD data but gave up because of challenges. | 1 | 1% |
| Other... | 9 | 7% |
| Working on project to pilot EHR-to-eCRF data exchange on clinical trials intended for regulatory decision-making | 1 |  |
| As Program Officer at a funder, I have overseen clinical research studies that included EHR, claims, and patient data. | 1 |  |
| Been involved in several initiatives looking at optimizing the use and reuse of various types of RWD for the rare disease field. | 1 |  |
| C-Path has used registry data to achieve qualification of a prognostic biomarker from FDA and EMA. | 1 |  |
| Collection of data from patients using mobile phone/web. | 1 |  |
| Fund projects that collect RWD. | 1 |  |
| I evaluate RWD study protocols submitted for regulatory decision making for drugs at the FDA. | 1 |  |
| I have reviewed and considered utilization of RWD for new drug application. | 1 |  |
| I was involved with an ONC/ASPE/PCORTF initiative to use RWD for evidence generation, Harmonization of Common Data Models (CDM). | 1 |  |

## Participants Experience with Data Standards

**Experience with implementing CDISC or other standards:**

| I have implemented CDISC standards outside of regulated clinical trials. | 18 | 25% |
| --- | --- | --- |
| I have reviewed data that uses CDISC standards as part of a regulatory submission process. | 15 | 21% |
| I have implemented CDISC standards for regulatory submissions. | 13 | 18% |
| I have heard of CDISC standards while implementing other data standards. | 12 | 16% |
| I have heard of CDISC standards, but I have not implemented or reviewed CDISC standards or any other data standards before. | 10 | 14% |
| Other... | [5](https://www.cdisc.org/node/172798/webform-results/analysis/19) | 7% |
| I have heard of CDISC standards and initiated a project to create NCI CDEs based on these standards. | 1 |  |
| I have participated in the development of standards, the Therapeutic Area Standards. | 1 |  |
| I used CDISCS data for analysis for the regulatory submissions. | 1 |  |
| We have implemented CDISC standards in our regulated clinical trials. The RWE data were not collected using CDISC standards. | 1 |  |
| Use of OMOP (Observational Medical Outcomes Partnership) common data model. | 1 |  |

**Sources of information that proved most useful in implementing CDISC standards**


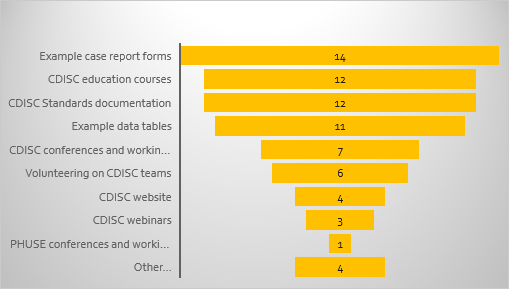


| Example case report forms | 14 | 19% |
| --- | --- | --- |
| CDISC education courses | 12 | 16% |
| CDISC Standards documentation | 12 | 16% |
| Example data tables | 11 | 15% |
| CDISC conferences and working groups | 7 | 9% |
| Volunteering on CDISC teams | 6 | 8% |
| CDISC website | 4 | 5% |
| CDISC webinars | 3 | 4% |
| PHUSE conferences and working groups | 1 | 1% |
| Other... | [4](https://www.cdisc.org/node/172798/webform-results/analysis/21) | 5% |
| CDISC User groups | 1 |  |
| Support from CDISC staff and volunteer teams. | 1 |  |
| Former member of the CDISC Board. | 1 |  |
| Worked on developing and implementing therapeutic area standards. The Tuberculosis was used in an FDA approval for a new drug. | 1 |  |

**Key benefits of implementing CDISC standards**

- The CDISC standard is robust and well documented.
- Enables the integration, storage, and later interpretation of large quantities of data, enhancing interoperability, retrieving, and sharing of information.
- Enables streamlining of the entire data life cycle - from creation of CRFs, QA and analysis, for a product that is ready for regulatory submissions.
- Allows for quality and efficiency gains - Data from multiple studies are more easily 'poolable’.
- Allows for a more unified language to be used within a team, with more focused discussions around organizing the data.
- Provides some consistency in data collection.
- All terms in the CDISC controlled terminology set have definitions.
- Standards are also the basis for automation.

**Main challenges in implementing CDISC standards**

- **Complexity**
  - Challenging to implement without much assistance from CDISC.
  - Insufficient and/or changing guidance on user guides.
  - Few implementation examples and sharing of experiences is difficult due to companies' privacy policies.
  - The standards can be quite complicated, especially SDTM.
  - SDTM is built for submission purposes, and not for RWD use.
  - It is not easy to map to SDTM.
- **Gaps**
  - The foundational SDTM standard does not always capture needed domains or concepts, and certain domains that are not easily adaptable to alternate uses.
  - Difficult to bridge between SDTM and CDE.
  - CDASH needs to be expanded.
- **Other challenges**
  - There is low awareness of the usefulness of sharing data, and it can be challenging to explain.
  - Implementers focus on data once it has been collected. The most important aspect for data quality is standardizing point of collection.

# Survey Results

## CDISC Focus and RWD

Participants were asked to comment and improve upon a draft diagram showing the major activities and sources of human health data and where CDISC currently fits, as well as and the potential broader focus areas of CDISC RWD Connect. The following diagram is the result of participants’ feedback (see below). The exercise proved useful to show that there is a lack of consensus on the definitions of RWD, its sources, and activities that generate RWD. Half of the participants strongly agreed with the final version of the diagram shown below, 42% moderately agreed, and 9% disagreed.


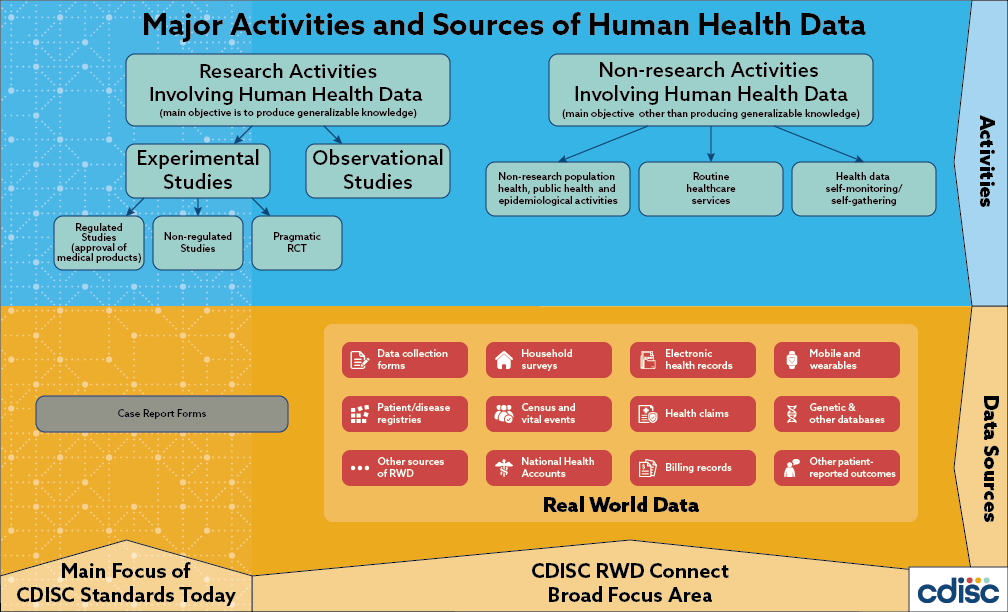


Notes from Authors:

- The prevailing view was that most RWD could be generated from any source, so we kept a single diagram and eliminated the arrows connecting activities and data sources.
- The diagram is an oversimplification of reality and it would be impractical to attempt to cover all possible sources and types of RWD. Attempts were made to accommodate all suggestions, some of which contradicted each other.
- The diagram was meant to generate consensus on the main types of data that are considered RWD and their possible data sources. The FDA defines Real-World Evidence (RWE) as “the clinical evidence about the usage and potential benefits or risks of a medical product derived from analysis of RWD.” Therefore, if we have consensus on the definition of RWD, then we believe the FDA definition of RWE can be applied.
- We agree public health activities can involve research activities, and those would then be included in the “research activities” on the left of the diagram. Research activities comprise activities using any kind of data, including public health sources, patient registries, etc. The diagram shows that there are some research activities and many non-research activities that generate RWD.

## Priority Components for CDISC RWD Connect

We asked participants to share which types of RWD CDISC should focus on first and why. Below is a summary of the participants’ answers after two rounds of revisions based on the feedback received. Of note, 62% of participants strongly agreed with the below summary of priorities and rationale, and 38% moderately agreed with them.

- CDISC should focus on **Electronic Health Records (EHR)** (25 responding groups) because they are one of the most readily available and largest data sources, they are already in electronic format, and they contain important and essential information directly relevant to the patients’ health status, and because they will be the hardest but the most important source of data for the generation of RWE. Another reason to focus on EHR first is that the data volume in EHR/EMR is rapidly growing and there is huge unmet need to standardize the data-processing and analytics, and because standards allow traceability and quality control, which is essential for acceptance by regulation. EHR may replicate some types of clinical trial as it records data such as labs and vital signs which are needed for monitoring safety and effectiveness in clinical trials, so there may be potential ability to assess effectiveness of a given treatment in a real clinical setting, although further investigation is needed on this. EHR already uses another standard (HL7) so we can convert easily using Biomedical Research Integrated Domain Group (BRIDG) model (ex. HL7 FHIR program is using ODM API and is mapping with CDISC CDASH and SDTM). CDISC needs to ensure that EHRs are connected to CDASH directly. CDISC could focus on harmonizing their standards with FHIR to establish an official set of mappings that are freely available in human readable and standardized machine-readable format for use to aggregate/map/transform data from different sources. Expanding the focus will severely dilute CDISC resources and influence because healthcare data standards are taken care of by HL7. No need for new and different standards in areas that are already covered by FHIR. The normalized Resources are a place to start in terms of which data elements to work on, but the harmonization is really with the owners of the concept systems used in EHRs and recommended in FHIR. There will still be institutional coding systems that have been in use for a long time and are not likely to change to CDISC. In the end, the owners of the concept systems will need to get involved if only to confirm the mappings. Start with FHIR resources to identify data elements that need to be harmonized because healthcare data systems are where most RWD arises, and HL7 has an active community to work with to do the harmonization work. EHR data is going to be increasing mined directly for research studies. Currently OMOP and other commons standards are being leveraged. CDISC should take a stand here on how data from Epic, Cerner, and other EHRs are harnessed for research.

Dissenting comments received on this point (2 responding groups):

- We should focus on the data used for regulated studies.
- Research, NOT EHR.
- **Observational studies** (11 responding groups) such as cohort studies, case control studies, and cross-sectional studies should also be focused on because they collect similar data to randomized clinical trial data, they tend to be fairly well structured already, they are far less developed in terms of standards compared with EHR, and the impact will be higher as largest proportion of data shared and reused is from observational studies. Also, because these studies are usually major methods in epidemiological and surveillance studies, and CDISC would map them well, although they sometimes do not cover the data collected in observational studies in the format that clinicians are used to seeing. OMOP is good for public health activities but CDISC is better than OMOP for research.
- CDISC should also focus on **registries** (9 responding groups) because they collect limited data, there is no consensus on how to store data and disease-specific concepts will map nicely to CDISC standards. Also, because their data may be used in combination with clinical trials. In parallel, CDISC should work to standardize their data sources such as EHR for efficient use of standardized data.
- Apps and mobile Health (**mHealth)** (7 responding groups) including mobile, wearables, genetic self-tests, could be an interesting area of focus, as the technological advances may make use of standards easier and more intuitive, and since they will be collected actively and passively for an increasing number of trials because they contain much data, a fraction of which may ultimately be usable. Start with mHealth data as the source where these devices are used in clinical trials.
- Mobiles/wearables should be a priority, as they are likely to be the most popular features of RWD used for research soon.
- **Billing records and medical claims data** (4 responding groups) could also be considered because they are often used as a proxy for health records, and both EHRs and claims are the RWD that will be submitted with regulatory submissions first. However, billing codes are a primary source of misinformation about a patient's health. Another caveat is that medical claim data depend fully on healthcare delivery system and health insurance system of each country, so it would be difficult to talk about international standards for medical claims. Claims data is in some ways a subset of EHR data. Therefore, CDISC should focus on a model for EHR data but keeping the other sources in mind to make the domains as reusable as possible.
- Before broadening the scope of CDISC, a **gap analysis** to gather insight into other standards stakeholders should be conducted (3 responding groups). There are already many established standards for some of the areas above, and often institutional standards as well. At a minimum to help aggregate and analyze data from these different systems, there could be **an official mapping between CDISC and other standard terminologies** that are typically used for the same data element. A mapping like this is very doable and given that most all other standard terminologies have terms that are not defined, this would improve those terminologies. Additionally, CDISC should focus determining how best to interface with other initiatives, such as CEDAR, that relates to (e)CRFs. CDISC should also focus on the **fundamentals of how to model and represent data**, and how to manage changes - unless this is done well, then building new additional standards on top of poor foundations does not necessarily bring any benefit. SDTM is currently more difficult to use than it needs to be, because some of the underlying principles are not well thought out (mainly in the areas of data types and data modelling).
- Academic-led **experimental non-regulated** studies (3 responding groups) because they follow similar clinical trial designs and their data is also useful to regulators in making public health decisions.
- RWD for **experimental studies intended for regulatory decision-making** and making sure these interoperate with HL7 FHIR.
- Prospective, randomized, **pragmatic trials** as these are most like the current Regulated Clinical Trails (RCTs) that CDISC standards are currently used for.
- CDISC should work to accommodate **all types of research data** into the standards because data are usually of higher quality than those collected in health records and are more likely to focus on health priorities. Public health data should be next as they have greatest potential to impact populations at greatest risk. These include other research studies (including population-based and translational research), surveillance systems, census and vital events, genetics, surveys, and other data collection instruments.

## Barriers to the use of CDISC Standards for RWD

Participants were asked what they thought were the most significant barriers to using CDISC standards in academic settings for RWD. A summary is below (we did not ask for level of agreement).

**Complexity** of the implementation of CDISC standards (32 responding groups)

- Common data models are commonly used for RWD and those are not usually CDISC. There are already existing standards for EHR, Claims, and billing data (ONC, FHIR, C-CDA, SNOMED, LOINC and ICD) and they prefer to use those standards and not introduce another standard. The primary challenges are therefore about harmonization, and adoption. It is not clear how CDISC could enter this existing landscape, but it could play an important role. The CDISC tools are not mature yet.
- Comparing to other standards such as ODISH and FHIR, CDISC standards seem to be more complex and require greater efforts to understand and implement. There is a need for complex reformatting/mapping and transformation to get RWD into the CDISC format, which is not easy or quick to learn.
- Academia uses different EHR systems at each institution, and it would be difficult to obtain the structured data that CDISC requires, due to the complexity of systems and their application to specific diseases domains.
- CDISC data are difficult to use for anyone who is used to analyzing wide, flat, data files (e.g. one observation per subject, with all information in one row), as CDISC data are long, narrow data files with multiple observations per subject. Once proper mapping and data dictionaries are developed, a good analyst/data manager can easily use the data, but developing the mapping and dictionaries takes time and expertise.
- The standards (primarily SDTM) are designed to fit a single clinical trial, including a set intervention and time. Real world health care does not work this way. The standards need to be adapted to accommodate longitudinal data, more flexible relationships (related persons) and a more diverse treatment regime without experimental therapies or where everything is an experimental therapy. SDTM presents challenges for the researchers because they say it does not cover their content or it introduces data reduction that can influence study results. Additionally, CDISC has variables such as trial design which are not needed for RWD, and there are variables important to RWD that are missing.
- SDTM is all about how to put clinical trial data into a SAS dataset. There needs to be a higher-level model for the data, by considering what are the entities being modelled, what attributes and properties those entities have, and how they relate to each other, and what the constraints are on the data. This could be developed side by side with existing SDTM standards and would map onto existing standards for as long as necessary.
- Academic organizations are interested in standardizing data at the point of collection because they realize this is where data quality is the most crucial. In one experience with an App, they were not able to implement CDISC from the start when building the database. Another group tried to incorporate it and because of their limited time and resources they decided to abandon the idea even though they thought it was of some value.
- Part of the challenge is the effort required to curate and transform existing RWD format to CDISC standards. Currently the data models used by researchers are different than how EHR or CDISC models data. Data for analysis is typically represented in flat files with discrete data elements for each data point of interest, for example HBC Result is the data of interest, then there is a column with this name, and each row represents an instance of the data point data elements that can be plotted/graphed directly compared and easily analyze. Statistical analysis packages work best with atomic data points formatted this way.
- Additionally, standardization is more likely to deal with existing terms, whereas academic settings often deal with new ideas or terms, so it is hard to standardize a term we have not well defined yet. There is a lack of agreed ontologies.
- CDISC standards were not developed for the observational studies, let alone other RWD. Additionally, the language clinicians use is not quite the same as CDISC. Clinicians who created the TA standards produced by HL7 and shared them with CDISC ask where their content was in the CDISC standard. And the RWD community will inherently value different elements of data, which makes implementation of a single standard less realistic.
- Flavors of null are very important in academia. Having that data available (no, unknown, n/a) all have different meanings, so they want that information in their allowable value sets.
- Requires investment in infrastructure across projects, e.g. for metadata repositories etc.
- There are privacy concerns.
- Inability to incorporate CDISC in the EHR.
- Other common data models are a better for healthcare data (Garza et all 2016 PMID: 27989817). With HL7 FHIR, healthcare data are now standardized at the data element / controlled terminology level, i.e., already standardized, and usable, nothing CDISC can or should add to this. Other common data models have already been mapped to each other and to FHIR.

Insufficient financial and trained human **resources** (15 responding groups)

- There are insufficient financial and human resources and expertise (budget, human resources, and available knowledge) and a low awareness on the academics’ side around the issue of data management. There is a general lack of understanding of the standards and insufficient capacity to implement them, with few experts who know how to implement the standards. CDISC is not used very much at all even in clinical trials in academia so the barriers are huge for RWD.
- There is a lack of training materials suited for academic users that are not IT experts.
- There are challenges related to working with academic IT offices.
- There is no information available for immediate implementation of the standards in academic settings.
- The cost to join/access CDISC standards.

Insufficient knowledge of CDISC Standards and their Value (13 responding groups)

- It is difficult to show the enormous value of using CDISC standards. People generally do not understand data standards and the value they bring, and they tend to think they are only able to add value for regulatory submissions. All explanations of data standards and how they work are too technical for decisionmakers. Often CROs provide the CRFs and EDC systems and the academic trial sites are not aware that standards are being used. Also, often in academia people think about datasets but do not really appreciate the distinctions between lists of data items or terms, and actual 'standards'.
- Medical professionals and researchers have a lack of awareness of the importance and value of data standards. There is not enough information about the potential benefits of CDISC standards for using RWD in academic settings. Additionally, academics have a long-established common practice of generating and collecting data without consideration of data sharing and standardization, and they suffer from dependency on current templates, formats, and software. Most academic settings already have a data collection system in place (e.g. EPIC), which would dis-incentivize the uptake of a standardization system.
- Additionally, there is a lack of cooperation by EHR vendors to adopt any standards, and there is push back from healthcare providers. And when an AMC learn about CDISC standards, they simply lack the motivation, know-how, finances, and experience to adopt them.

Lack of real and perceived **incentives** for using CDISC Standards (6 responding groups)

- There is a lack of a perceived benefit of using CDISC standards for RWD analysis, beyond FDA reporting, so academics do not see any incentive to make the extra effort. The direct overhead to the investigators is higher than the perceived benefit.
- CDISC standards are not a requirement for publication of results. In the world of academics, often the main purpose of their effort is a publication, and standards not yet enforced for publishing, although this is changing.

Other barriers (2 responding groups):

- For RCTs that have already reported and are no longer recruiting, retrospectively converting all such data to CDISC SDTM would probably not be feasible given the significant resource this would require.
- Decentralized nature of academic decision making (i.e. hard for institutions to impose a standards policy across multiple different groups).
- The retrospective conversion of prospective studies data would be challenging.
- Data collection and the creation of a study database for many prospective studies is carried out in collaboration with other institutions (in other countries) for whom CDISC is likely not standard and where the infrastructure to adopt it may not exist.

## Steps Needed for Implementation of CDISC in Academia

We asked participants what they thought it would take to achieve implementation of CDISC standards across or within academic institutions. Here are their responses:

**Adapting and improving the standards and supporting tools and materials** (15 responding groups)

Standards themselves:

- There is a need for improved CDISC standards which are more informed by current knowledge and practice in computer science and data science and developed in collaboration with academic institutions. Engaging research communities to collaboratively develop the standards, especially when linked to an initiative on core outcomes, would promote acceptance.
- More focus on standardizing at point of collection or patient care, since SDTM does not consider data variations at the point of collection that can influence research results. CDASH has the most value for standardizing at the point of care.
- Better integration with HL7 standards is critical.

Implementation guides:

- Easy to use implementation guides. There is a need for specific implementation guide for observational studies, which will be a huge work, because observational studies have more freedom for its metadata compared to the interventional studies.

Support tools:

- Implementation for academics should be as easy and automated as possible to reduce the burden of standardization.
- Standardized research tools are needed (case record forms, database templates) that have the standards built in. These should be simplified, easy to use software tools with machine readability of specifications to make implementation much easier. They would need to be open-source/accessible for free and have good user interfaces that draw on standard ontologies that can be used by non-CDISC experts.
- Academia cannot afford to create a conversion program from CDASH to SDTM. If possible, there should be no coding tools.
- We also need multiple pre-existing templates with standards defined, and an EDC template that can be quickly implemented in an EDC.
- Additionally, there is a need for good quality language translations of the standards, educational materials and user manuals conducted by native speakers.

**Providing funding and incentives for its implementation** (10 responding groups)

- Provide financial incentives for implementation (like meaningful use).
- Free CDISC membership for medical institutions.
- Seek grants for CDISC to train academics or grants for academics to apply for directly to implement CDISC standards, including data curation.
- Make the value proposition for its use for research clear, beyond the FDA reporting requirement, in order for researchers to demand the use of CDISC standards by the two groups who would need to implement the standards: software developers of off-the-shelf products like Medidata Rave, and individual researchers who design their own databases into which they extract and reenter data.
- Make the case for CDISC standards to increase patient safety: Broad adoption of CDISC standards would ease the development and implementation of automated adverse event reporting and better protocol deviation detection.
- Make the case for increased appeal for pharma funding: The implementation of standards might make the academic medical centers more attractive to a pharma looking to open a trial.

**Sharing of experiences and use cases** (10 responding groups)

- Communicating and showing the concrete value of embracing data standards could be done through a communication campaign, including case studies. CDISC should provide examples and use cases to demonstrate how CDISC data standards can benefit academics, their institutions, their research, and teaching work, and how it can benefit health research globally.
- NIH and other funding agencies should provide concrete opportunities for knowledge and experiences sharing within and between academic institutions for efficient use of CDISC standards for RWD.

**Regulation and policy changes** (8 responding groups)

- A mandate from funding agencies to use CDISC standards for the types of academic research that best aligns with CDISC standards, including registries or EHR data. Standardization of protocol requirements, and mandatory requirements to comply with data interoperability standards for grant-funded research. NIH, for example, could use their grants to encourage the use of CDISC standards within different institutes or across academic institutions that have their own systems to collect RWD, making implementation of CDISC standards a condition of funding.
- Support from EMA / European Commission / other national regulatory bodies for the use of CDISC standards for certain purposes, for instance: a whole health system embracing CDISC standards.

**Providing affordable or free training opportunities and support** (7 responding groups)

- Trained data managers, statisticians and Principal Investigators (PIs) who can adopt the standards are needed. There should be training opportunities provided at an affordable cost or even for free to academic users, including educational efforts such as webinars, tutorials, and open source data transformation implementations, including lots of data support, working with the academic institutions (e.g. mentors). Create a suitable business model that works for potential academic non-expert users.

**Champions** (4 responding groups)

- There is a need for far-sighted leaders who know about the enormous value of using CDISC standards. A champion from a prominent academic institution could be identified for CDISC implementation.
- The EHR data from one of the prominent EHR companies (Epic?) could be mapped to conform to CDISC and those data become available only in the CDISC format. Then, academic institutions or other groups wanting to use those data, will need to learn CDISC. Once they are familiar with CDISC data, conformance of their own data to CDISC may be more palatable.

**A dedicated group or service within institutions** (2 responding groups)

- Anticipate that each institution/group would require a dedicated data individual or standardization group to map study data to standard variables. There could be a special implementing service with certified teams.

## Making the Case for Using CDISC Standards for RWD

We asked participants what they saw as the main challenges in academic clinical research that could be overcome with increased standardization of RWD, their responses included:

**Different sources of data and poor integration and interoperability**

- All the different sources of EHR and how its entered. Large text fields of physicians’ notes make interoperability difficult.
- Poor integration of data across different systems and poor interoperability.
- Data interoperability - data exist in many systems that do not speak to each other well. Much time and effort are spent getting data from one system (e.g. a Laboratory information system) into another system (e.g. some registry for public health research).
- Inconsistency in the way the data is collected, different variable names and permissible values, poor data quality
- Data silos. Everyone is in theory keen to share/pool/be able to query data - but nobody has collected source data in a standardized manner, thus it becomes an insurmountable challenge to try to pool it or increasingly, link it, for any sort of reuse. People are keen to avoid duplication of data collection/entry.
- difficulty in data reuse
- Right now, most data collection of RWD is performed in bespoke ways. Data from the EHR is collected and transformed into a variety of standards like OMOP, PCORNET, or often, no standard. The end-user is left to develop new analysis tools to leverage these non-standardized data.
- Data are collected in non-standardized ways using CRFs and other data collection forms that do not leverage standardized vocabularies. By standardizing RWD collection, PROs could be developed using a consistent data dictionary, thus easing the analysis of the data, and promoting better interoperability.
- Data from wearables are completely non-standardized and at the mercy of the manufacturer. Bring Your Own Data (BYOD) data has been transformed and manipulated many times by the vendor prior to analysis. Metrics such as sleep quality or average daily heart rate are not defined. Data transformation on devices is not documented.
- Institutions do not have time, skills, or incentives to develop their own internal comprehensive standards. Data modelling problems are currently solved in a different way by every group within every institution.
- Archiving and long-term storage of data are also areas that a lot of institutions struggle with, because short term tasks are always more pressing
- Time and effort to map and transform data. Any time data is mapped or transformed, there is the potential for loss and/or errors and results may not be trusted.
- High efforts of merging data.
- Data from clinical information systems might not be interoperable, due to the lack of standardization.
- Inconsistencies in data (missingness, poor coding, rich clinical data left uncoded as narrative text).

**Other challenges:**

- IT department in hospitals only supports routine medical activities but not research activities.
- Cost of conducting meta-analyses which are currently high due to having to curate and standardize heterogenous datasets.
- Researchers need to analyze multiple studies and subsequently derive conclusions.
- There is an absence of harmonization of clinical trial initiation and conduct across academic clinical research sites. This is largely driven by the fact that different sponsors often impose myriad requirements on academic clinical trial sites. Expectations for clinical trial site performance may vary by sponsor, and even within a single sponsor. Verifying the quality of the research being conducted at a site means multiple and often duplicative on-site visits. These can be time consuming and costly, yet there is no evidence that this level of on-site monitoring improves outcomes.
- The people in the academic research exhibit a lack of need for standardization unlike in pharma. Do not link academia to Real World Evidence (RWE) as a 1:1.
- Very few. FHIR will help greatly by enabling extraction of EHR data and population of EDC systems or CTMS systems.
- Meta-Analysis of Individual Participant Data (IPD) from RCTs and other types of meta-analyses.

## Tools or Support Needed

We asked participants what tools or support would help to standardize and leverage RWD. The responses focused on providing templates, standards and User Guides, and on robust software tooling to enable efficient data collection, mapping, integration, transformation, and analysis tools as well as common data models, dictionaries and quality control/validation and finally education and training.

**Templates**

- Both standards (CDASH CRFs) and software.
- Providing template or format for data collection.
- Availability of pre-made annotated CRF for all medical fields.

**Software and Standards**

- Computer software that can standardize the electronic records and information across different institutions.
- CRFs and database ODM files with CDASH and SDTM coding built in.
- FHIR (Fast Healthcare Interoperability Resources
- Research software and systems that use the standards
- Open source easy to use tools, Flexible tools, Web-based tools
- Data Collection Tools
- Computer software that can standardize the electronic records and information across different institutions.

**Mapping, Integration and Transformation Tools**

- Tools for mapping to the standards.
- Mapping documents and data dictionaries
- Mapping and transformation tools that use the standards as metadata to help streamline the process.
- Tools that auto map to standards so that a clinician or researcher does not have to think about it. The best is not having to map. Use a healthcare standard and use CDISC for research specific content. It must be mapped at the point of data collection during a healthcare encounter...built in to the Electronic Health Records (EHR) tooling.
- Tools for data transformation
- Tools for data integration

**Analysis**

- Tools that allow for close integration with tools like R
- An online tool to help researcher to convert to standard (many companies use SAS codes to convert their data to CDISC standard). JMP Clinical has an add-in to convert to standard one domain at a time without coding. This JMP Clinical add-in is for the analysis purpose but can serve an example as simplified tool for implementation.
- Better analytics for mining RWD

**Standards and User Guides**

- FHIR (instance of above bullet point – focus on content standards).
- User guides on how to analyze CDISC data.
- Clear guidelines on how standards are implemented.
- Implementation guide, specific for the observational studies.
- Easy documentation for end-users setting up studies.
- Standards for the wider range of RWD including observational studies.

**Common Data Models and Dictionaries**

- Common data model and data dictionary.
- Clinical vocabulary management.
- Common terminologies and accompanying methods to harmonize terminologies and transform one terminology to another.
- Standard code for laboratory tests.
- Data dictionaries focused on particular diseases that act as cheat-sheets for data managers setting up Case Report Forms (CRFs) and databases.

**Quality control/Validation**

- Common quality control criteria.
- Analysis tools to check the quality and conformance of the data as well as analyze the data without the need for custom programming tools.
- Focus on improving the quality of RWD.
- Terminology and metadata validation tools, open-source Export Transform Load (ETL) tools.
- Change control, feedback on examples of use.
- In observational research, the use of data can give instructions directly to the server and return only the results. A data management program to confirm the same quality can be executed.

**Education**

- Education as to the why is needed to create a pull from researchers (to use CDISC standards).

**Other**

- A way to help academics use standards in their existing resources (ideally without having to do away with the registries/systems they already use, but instead make these better, more robust).
- Library of standards that is easy to understand.
- Regulator-approved methods of integrating, coding, and analyzing RWD for regulatory submission Infectious Disease Data Observatory (IDDO) is pursuing this with European Medicines Agency (EMA)).

## Benefits and Opportunities from Standardization of RWD

We also asked what participants saw as the primary benefits and opportunities from standardization of RWD, and specifically, how they would make this case to their colleagues. These were their recommendations; 80% of participants strongly agreed with this revised summary of the benefits and opportunities from RWD standardization:

**Data sharing:**

- A common data model means no additional data mapping is necessary when data are coming from different sources.
- Mapping and transformation will not be eliminated, but the effort could be reduced with the availability of standardized mappings and transformations between existing standards, available in standardized formats for machine use.
- Sharing across institutes of the data.
- Data sharing and collaborative work among academic institutions or with industry, and use of research results for supportive information of drug/device approval
- A common language would make pooling of data easier.
- Increased statistical power in neglected diseases, where data volumes are low. More representative evidence that can be generalized across a broader population.
- in the Rare Disease field - all data is precious, as there are so few patients with any single rare disease, so it is only by pooling or somehow linking the data that does exist (often from different sources e.g. registries, trials, EHRs, biobanks etc.) that you can attain a critical mass and advance knowledge and care.
- Ability to scan many studies to consider whether they can answer new scientific questions would be helped by machine readable data.
- Test the results of clinical trials in a real-world setting, that could confirm the utility of a treatment in all patients for daily clinical use, and perhaps search for a personalized strategy of treatment.
- If all data were in the same standard, much time and effort could be saved, and analysis would be faster.
- Standard vocabularies.
- Use of standard terminology so there is no ambiguity to interpret.
- Cost reductions for research via decreased effort for data management.
- Consistent statistical analysis.
- Data modelling problems that are currently solved in a different way by every group within every institution could instead be looked up in a domain-specific standard.
- Standardization allows automated extraction of data, which would enhance productivity of researchers significantly.
- To facilitate the synthesis of data from different sources,
- Have an impact on health outcomes, basically makes it easier to share data.
- Integration of research and non-research health data.
- Use of standards is an essential cornerstone of the FAIR data principles (https://www.force11.org/group/fairgroup/fairprinciples).

**Improve quality of research:**

- It would improve the accuracy of null hypothesis prior to conducting a clinical trial, increasing the probability of success in drug development.
- Improvement of scalability for research. If all research institutes use standards, larger amount of subject data will be used for research, which increases the quality of the evidence.
- Promotion of new ways to do clinical research in a larger scale.
- Increased standardization of RWD would allow implementation of more research studies that utilize RWE, especially for regulatory decision making.
- Carrying out metanalysis on a single dataset as opposed to a query system in which researchers analyze multiple studies and subsequently derive conclusions.
- Better data around research on health and longevity
- Quality control and assurance.
- Automatic extraction data from EHR would not only reduce the burden on clinicians but also would allow for bulk data extraction, reduce errors in transcribing and selection bias.
- By doing RWD standardization, sponsor will have the chance to better understand their RWD data and to improve/optimize their original study design.
- Being able to marshal RWD into RWE requires accurate data. It will require a lot of data (terabytes!) to draw accurate results. Achieving accurate results requires a common language, harmonization, and codified and structured data. We need standardization to achieve the promise of RWD.

**Other:**

- Creating standards for the use of mobile data in clinical research - especially for BYOD - would help stabilize the industry - at least the companies that seem to care about clinical research (e.g., Garmin).
- Increasing consistency in clinical trial initiation and execution could reduce costs, increase data integrity, speed development, and better serve research participant. Standardization of data (and RWD in particular) would likely need to take place in this broader context of standardization/ /harmonization across academic clinical trial sites.
- International comparison between claims databases.
- Standardization would help with archiving and long-term storage of data because it reduces the amount of documentation needed before archiving.
- To standardize the way that data collection tools are developed for multiple studies
- Speed up the production of high-quality papers. RWD-based evidence generation will be accepted by regulators and journals with more confidence and efficiency.
- Standardization of RWD in a regulatory submission to better support RWE. Standardized RWE will improve data clarity, data transparency and data integration if FDA reviewers will receive the RWE in their BLAs, INDs and NDAs in future. As an example, in each of the subgroups of the HMA-EMA Joint Big Data Task Force, i.e. for each type of data (Clinical Trial and Imaging, Observational data, Genomics, Spontaneous Adverse Drug Reaction, Bioanalytical Omics, Social media/m-health) the need for standardization was raised as a key prerequisite in order to drive harmonization across datasets, enhance interoperability, improve data quality and facilitate data analyses. See reports of the HMA-EMA Joint Big Data Task Force: https://www.hma.eu/509.html#c5635.
- Reproducibility over time. With standardized data, it is much easier to create data collection tools and analytic programs. they can be made once, and with few modifications for future studies and to allow for new innovations, then used again and again for future research. The ramp-up time to creation of analytic programs is minimized and the time to results is much faster.
- It may require an initial investment, but at the end, it may save money and time. Standardization will directly result to more output from a given dataset, reduced costs as the data does not need to be collected again and increased efficiencies as data will be in a format that is widely understood.
- Issues of ethics and privacy protection can be addressed more strictly.
- For long-term follow-up of a clinical trial (e.g. 15 years required for some gene therapy trials), the use of registries and other RWD will be essential in providing the information needed to assess both the safety and efficacy of the treatment.
- For each disease of interest, development of CDISC-compliant data tools would involve (briefly):
  - Identification of key stakeholders to participate in the development and review, including regulators, pharmaceutical actors, academia, and local/international organizations providing treatment.
  - Collation of diverse data sets to understand the scope of variables required.
  - Drafting of case record forms (CRFs), informed by historic datasets and discussions with regulators on the data requirements for future license applications.
  - Circulate the draft CRFs for wide stakeholder input and/or organize a stakeholder meeting to achieve consensus on final content. The engagement of these stakeholders offers additional opportunity for rallying consensus on outcome measures and statistical methods for further streamlining of drug development, regulatory submissions, and post-marketing research.
- Contributing to the Learning Healthcare System.
- Long term storage format for observational research using RWD. Explanatory factors can not define completely at the start of the observation, and within the study period, some factors added to the database.
- For stakeholders interested in conducting clinical trials in rare diseases or neglected populations, it is often difficult to know what the 'standard-of-care' is, and that standard of care is what should be used as the control arm in any trial.
- It is necessary to consider the advantage to the vendor because it leads to the disadvantage of the vendor such as electronic medical records.

**Comments from participants (n=2) did not agree that there were benefits and opportunities from RWD standardization.**

- Currently, there is no advantage gained from the standardization of RWD, but we are considering standardization of the collected contents. I believe that standardization will benefit in the future.
- RWD cannot be standardized. We (research) are secondary data use and as such are the tail wagging the dog. We small researchers cannot (and should not try to) standardize healthcare data.

## How to Build Knowledge and Expertise on CDISC Implementation

We also asked about what the most effective ways are to build knowledge and expertise on implementation of CDISC standards in academic institutions. Responses included:

**Provide funding for capacity building**

- Give grants to academic institutions.
- Fund institutional roles that can support researchers to implement.
- NIH to give out grants to research institutes.
- Additionally, CDISC to get involved in meetings where research investors are present.
- Funding from sponsors/funders.
- Researchers are awash in a sea of sponsors, with multiple requirements and competing standards, therefore some altering of the landscape through incentives is needed. Foundationally, it requires leadership, cooperation across institutions and an up-front investment of time and resources.

**Collaboration with, and among. institutions**

- Collaboration.
- Get together, talk and compromise.
- Build a network of users
- A small group face-to-face, hands-on workshops held in a local language, under reasonable pricing.
- Identify champions who will act as vocal leaders in different fields to educate colleagues and advocate for the benefits of adoption (5 responding groups).
- Engage with academic institutions and networks (e.g. UK CRC registered CTU network).
- Invite them to the CDISC Interchange meetings
- Promote CDISC via medical and research professional associations.

**Provide training certification mechanism**

- Train the trainers regularly and give certification.
- Set up a special task force team that functions as the trainer of the trainers. This team should have deep knowledge of CDISC and experience in implementing that in academic environments.

**Develop training tools, particularly online options**

- Make training free and accessible - both online and in-person.
- Establishment of a portal site to share their experiences and best practice, and to provide useful tools or templates
- Courses and open source tools.
- Set up workshops and webinars to support remote study.
- Set up on-line courses that people can study within a period and get the certification.
- Work with innovative universities and do pilots.
- More templates- CRFs, data dictionaries, datasets (based on real world scenarios)
- Training classes, including web-based modules.
- Make education/training tools available to those that are leveraging the data - the CRAs, analysts, etc.
- Wiki type open website and eLearning materials might be easier on academics.
- Build the standards into the systems used by researchers so the researchers themselves do not need to learn them, they would just be how the data was represented and they would work with it.
- It needs to be simple!

**Document experiences through use cases**

- Training including successful implementation cases of CDISC standards.
- Build up use cases for academia, to help researchers and investigators easily understand the benefits of CDISC data standards.
- Set up demo cases.
- Campaign based on use cases to show key people at all levels (key decision makers right down to actual data handlers/collectors) the value of standards for THEIR work.
- Pick a few leading studies and promote them publicly as exemplars
- To put as much samples as possible on the public domains. Samples mean a set of a clinical study protocol, annotated-CRF and define.xml.
- Describe the standards that were used in Methods sections of papers and in methodology publications.

**Provide on-site training**

- Offer training on-site in academic institutions.
- More outreach trainings.
- The right people need to attend the courses (e.g. those involved in developing data collection tools, analysts, statisticians).
- On-site training is a necessary action.
- More training opportunities focusing on 'how to'. Training to be tailored to expertise level and to include more hands-on exercises.
- Training on CDISC standards, mainly PRM and Clinical Data Acquisition Standards Harmonization (CDASH), in medical institutions.
- Targeted training and education. Data warehouse analysts, data modelers, CRAs, clinical trials office. The sale needs to be made at several levels - targeting clinicians will ultimately not help.
- Attend their networking or professional meetings to train.
- Provide training programs to the interested individuals in academic institutions, supported with good online documentation and courses.

## How to Reward and Promote the Use of CDISC Standards in Academia

We asked participants what the best way is to reward or promote the use of CDISC standards in academic settings, and here are their suggestions:

**Strategies to** reward **the use of CDISC standards:**

**Financial rewards:**

- Provide grants to consortiums implementing data management
- Monetary.
- Get funders to permit separate budget lines for the implementation of data standards.
- Funding incentives.

**Formal recognition:**

- A dedicated section on the CDISC website for the recognition of contributors and implementing institutions is also necessary.
- Publish a CDISC list of champion institutions.
- Recognition.
- Credit and recognition in future funding applications.
- Working closely with researchers to provide them the support they need to standardize datasets, then highlighting these cases as success stories publically.
- Have a show case
- Propose having connectathons for researchers in academic settings and award them for the best use case. Like challenge programs by HHS.
- Acknowledgement
- Build a CDISC certification program

**Publication:**

- Provide opportunities for helping to write up and publish methodological research papers
- Publish papers of high quality.
- Research papers is the most important reward. The CDISC society should be aiming at leading or helping the researchers to deliver studies based CDISC standards.
- Publication incentives.
- Get into relationship with journals to promote the use of CDISC standards in papers as an incentive to publication.

**Other rewards:**

- Other type of incentives to adhere to the new standardized system
- Efficiency will be the reward eventually.
- Approval of any new medication or an expanded indication would also be a very important reward.

**Other comments:**

- I think the introduction of more standards to an already crowded landscape will not be helpful. Leadership and vision are required to achieve true harmonization among existing standards. CDISC has an established footprint in regulated research landscape and seems poised to have a larger presence in academic research. Short of a statutory or agency requirement to use CDISC standards, incentives will be required, at both the institutional and researcher/PI level.

**Strategies to Promote the use of CDISC standards:**

**Get buy-in from funders and other key stakeholders:**

- Convince funders to fund CDISC development, implementation, and promotion activities.
- Target those who do meta-analysis, including Cochrane, to promote standards as it would greatly reduce their workload.
- Engage data repositories in the promotion of standards.
- CDISC can ask about the process used in each academic institution and what they see as pain points and try work with them in collaborative ways.
- Funding through NIH and foundations' grants.
- Providing grant money to facilitate the use of standards
- Engage funders and ask them to make recommendations to their investigators that CDISC should be used in their studies
- Increasing awareness about CDISC standards among journal editorial boards. If you educate journal editors in understanding that the use of CDISC standards improves the quality of research (and ultimately aim to get these standards endorsed by major journals), researchers will be rewarded when they refer to the use of CDISC standards in their publications, because this will increase the chance that their manuscript will be published.

**Regulation:**

- Regulations.
- Enforce the use of standards as part of the funding mechanism and institutional requirements
- When NIH funding requires it, researchers will follow.

**Publication of use cases:**

- Building up use-cases.
- Produce papers of high quality that make the case for the use of CDISC standards.
- A statement/position paper about CDISC-RWD will be also a great strategy to promote more professionals to join.
- More success stories=more confidence and certainty that the effort is worthwhile. Include: the number of extra citations, number of papers from this dataset, quicker impact on policy, amount of time saved, and amount of money saved.
- Demonstrate value to answering a question across multiple data sets (organizations) that cannot be accomplished as well not using standards. Show how it can change practice, treatment or reduce costs.

**Provide affordable trainings and tools:**

- More affordable training.
- Setting up a portal site for experience sharing and providing tools/templates
- Establish a data repository which are curated in CDISC standards and allow researchers to access after application and approval, a model like the one CancerLinQ adopted
- Make tools specific for academia to use CDISC standards.
- Free tools for the use of CDISC standards of RWD.
- Make sure everything is free to use and open source
- Promoting the idea of a shared data resource (like the NIH Data Common idea).
- Promote at international conferences through methodology sessions and make the session titles simple and not too full of jargon for those not familiar with CDISC.

## Standards for Devices and Wearables

When asked for their thoughts on the most significant challenges related to implementing data standards for innovative data gathering technologies such as consumer wearables (e.g., Fitbit, Apple watches, cardio monitoring), participants replied:

**Reliability of the data gathered by the devices**

- Credibility of the gathered data themselves.
- Accuracy and reliability are still a concerning issue in mobile and wearables.
- Proprietary algorithms that have not been clinically tested. Large RCT should be conducted on them. Clinicians should prescribe to their patient’s wearables or apps that have been clinical tested. Right now, vendors are making money marketing directly to consumers, so they do not have to meet this requirement. This may change if insurance covers apps that have been tested and may comply with certain healthcare standards. This may be the only place where mapping (algorithms to a standard) may be acceptable.

**Lack of incentive for device manufacturers**

- Multiple profit-driven providers with no incentive to standardize
- These devices are already on the market and already have their proprietary data standard that may be difficult to change if they have analysis and data sharing already built in.
- Getting buy-in from providers.
- Standards are unknown unknowns for some organizations.
- The companies producing consumer wearables may already have their own data standards.
- Challenge to convince and include different vendors and let them see the potential.
- Convince the technology companies that they need to do this to benefit patients (interoperability with EMR data and other systems) and themselves (standardized data will drive better innovation and more business).
- Alternatively, convince the consumers to put pressure on companies. Tell the consumers the benefits of data standardization.
- The only vendors that will care are those that are interested in getting into clinical research (Garmin, perhaps Fitbit now that they are bought by Google). Once a standard is published and adopted, the vendors that care about research will/should adopt it for their wearables.
- The standards need to structure in a way that make sense to software developers and can be directly used by them, there needs to be a perceived benefit, and they need to understand how to implement it in their systems.
- Right now, those types of developers seem to be looking at FHIR and the SmartOnFHIR API. That said, the contents of the FHIR messages could potentially carry data using CDISC standards - if the data elements in the FHIR Resources are modeled the same way for fields as CDISC modeled the field.
- Some organizations may have lock-in as a business model, not sure how to deal with that.
- No clear requirement for standards uses from FDA CDRH.
- Lack of regulation, no need for regulation.

**Interoperability and interpretability issues**

- Challenges related to data infrastructure, namely that there are no well-developed standards that would help to organize, annotate, and standardize the data and provide data mapping tools to electronic data capture (EDC) databases. The lack of mobile technology data standards is exacerbated by the fact that wearable devices sometimes report variables pertinent to the same phenomenon (e.g., mobility) but use different terminology, and data processing algorithms are not disclosed. The solution should include industry-wide standards for data and terminology, processing principles for similar sets of data, and transparency requirements around data processing algorithms. This seems like an area that could benefit from CDISC's involvement.
- Data structures are not transparent.
- Understand whether they are interoperable (meaning whether a heart rate of 80 in Fitbit also means a heart rate of 80 in Apple watch).
- Inadequacies or deficiency in the interoperability/communication among the different operating systems (ex. Android, iOS, or Windows) being used in RWD data collection.
- Propriety algorithms for different manufacturers.
- Data format and data input to analysis tools.
- Since amount of data is huge, it is important to implement data standards at data collection stage or at early timing of data collection and storage process.
- Some raw data is open to the user, but many devices cannot export the data. These raw device data are difficult to understand due to their complexity, so tools are needed to understand the data.
- How can data from consumer-driven devices actually 'speak' with for instance GP systems, EHRs, etc.
- The data was not generated for the research purpose, the significant heterogeneity within and between data sources might be almost impossible to overcome (how the data was captured, the precise semantics of the data, types of data collected, lack of reliable metadata, etc.), a lack (for now) of well-established methods of data curation and data quality assessment.

**Privacy and equity concerns**

- Concerns over ownership of data and who will access the data.
- It would also be difficult to obtain appropriate consent from participants, especially for those who are elderly, either due to difficulty to obtain e-consent, or the fact that few people use wearable devices.
- Sometimes going digital means leaving populations out of the picture. It can help some populations participate in healthcare and research, but others may not have access (data plan, cell service, internet service).

**Continuously evolving nature of devices**

- I think the continuous nature of wearables make this a challenging area to institute data standards.
- Devices evolve very quickly - hard to keep up with the time needed to develop community standards.

**Insufficient experiences using data from devices**

- Lack of concrete use cases that show who and how wearable data can be used for which purpose.
- Only experience of wearables is in a very regimented clinical trial and it was very problematic.

## Patient's Perspective in RWD

We also asked how participants would recommend that CDISC include the patient perspective in this initiative; they provided the following suggestions:

**Involving patients’ voice in the discussion around health data standards**

- The patient's perspective could be useful to gain insight into the utility of RWD.
- It is also important to have the patient-oriented value to the picture of developing standards because the mission of FDA is to protect public health including patients' health.
- Patient involvement should relate to the kind of data that should be collected, not how to standardize data.
- There is no benefit in including patients’ perspectives regarding whether CDISC is useful to the collection of RWD.
- Some of the talk and work around data standards may be technical/wonky, but patient involvement should be anchored in efforts to ensure that the widespread adoption of data standards will: (1) enable patients to get copies of their record in useful, digital formats, and (2) that the vast amount of data collected by electronic medical record systems will be used to help clinicians make decisions about patients’ care (which is not the case at the present time).
- Ask the professional organizations on the best way to include patients from various walks of life. NCATS may be able to connect to CTSAS, and networks (rural and pediatric). Seek populations who may and those who may not be digitally savvy. Ask if they are interested in using their devices for research, their barriers, and benefits.
- Patient privacy is a big issue. Discussion should involve patient advocacy groups.
- Select patient advocacy organizations should be tapped for input.
- Having patient advocate representation at all levels/stages is essential.
- The perspective of the patient would be helpful. They would support interoperability of their health data and are very supportive of sharing so by proxy would support standards
- Patients should be polled about potential ways their data will be collected and used and be given the opportunity for feedback.
- Consider systematic involvement of patients and patient organizations in the project (e.g. advisory council, specific work packages).
- Invite patient advocacy groups to participate in the development of standards. Be inclusive.
- Inclusion of patient representatives in steering committee or workshops
- Use established clinical medical society, and specific disease foundations to outreach to patients to provide input on this question.
- Conduct focus groups and involve them in usability studies.
- Invite representatives from patient advocacy organizations to the CDISC RWD initiative.
- Include patient representatives (with relevant experience) among advisory panel for all aspects of data standards development/implementation.
- Create a patient advisory group (including some representatives from areas like rare diseases where data standardization would be particularly powerful); include some patients in your EAB directly.

**Standardizing data that is most relevant to patients**

- This initiative should piggyback onto methods and activities which have established patient-engagement activities (such as core outcome measure initiatives) and make data standards the added bonus.
- Patient reported outcomes (PRO) have gained a lot of attention recently. We should standardize PRO data model and measures.
- Patient-reported outcomes (PRO) are an important source of RWD. Need to address the unmet needs and operational barriers with patient when designing that part of standards.
- Involve the current alliance that is promoting PRO.
- Consider including the following different data types in the RWD definition (see question 10 for references): patient preference data or patient preference information, patient relevant outcomes, patient reported outcomes, patient experience data. It could be discussed how these different types of data relate or differ.
- To prepare for the new era where clinical data and patient generated data are integrated, and researchers use RWD to derive RWE that would answer questions patients would like to know, this initiative may want to keep track of such area as tele-health, mobile-health, and how standards could help facilitate deriving RWE for patient’s illness.
- Consider the need to create data quality standards specifically for these different types of data/studies. Consider aligning efforts to build requirements with the following related projects:
  - IMI PREFER: will develop recommendations on the design and conduct of patient preference studies https://www.imi-prefer.eu/ , and have published several papers on project findings so far: https://www.imi-prefer.eu/digitalAssets/816/c_816010-l_1-k_digital-imi-prefer-publications_final.pdf . Several of their findings could be applicable to RWD quality requirements
  - IMI PARADIM: will produce a framework for structured patient engagement https://imi-paradigm.eu/
  - FDA patient focused drug development program including a COA Pilot Grant Program to support the development of publicly available core set(s) of Clinical Outcome Assessments (COAs) and their related endpoints for specific disease indications: https://www.fda.gov/drugs/development-approval-process-drugs/cder-patient-focused-drug-development
  - FDA draft guidance on patient experience data https://www.fda.gov/regulatory-information/search-fda-guidance-documents/developing-and-submitting-proposed-draft-guidance-relating-patient-experience-data
  - IAHPR: is currently writing a book, a how-to guide and teaching tool, listing the major decisions and crucial steps involved in developing and conducting survey-based, health preference studies http://iahpr.org/collaborations/
  - EUPATI: provides education and training to increase the capacity and capability of patients to understand and contribute to medicines research and development (R&D) https://www.eupati.eu/

**Educating patients on how data standardization benefits them**

- Engage the public to explain that putting patient data into a common standard facilitates comprehensive analyses; in turn this should help with generating reliable evidence which will lead to better informed clinical guidelines and patient care.
- If CDISC includes the patient perspective, the education and distribution of information to patient is a key issue.
- Patient groups should be educated (where necessary) about the benefits of standards as they can be strong advocates for this type of health accelerator.
- Many people do not share data because they don't know what it is used for. Increase patients' awareness of the usefulness of the data for themselves and for knowledge generation.
- CDISC should build use cases to explain how RWD standardization relates to patients' satisfaction and improve their quality of health care.
- Show that data standards could provide a way for patients’ data to be used to provide better healthcare.
- The potential perspective is the realization of personalized and precision medicine for patients. There is a lot work that needs to go into algorithm development to this vision to be achieved, for which data standards are needed.
- Hold forums to educate patients about standardization and what it means directly for them. Patients are increasingly becoming more aware and taking more ownership of their data. Discussions around use of blockchain for health data are a driver for use of data standards.

## Collaborations with Other Standards and Initiatives

Leveraging RWD, CDISC and other established and emerging standards (e.g., HL7-FHIR, BRIDG, OHDSI-OMOP, Sentinel, etc.) have the potential to transform the healthcare industry by focusing on each their strengths to deliver a holistic, interoperable future state that, will foster greater efficiencies across systems and resources, and encourage end users to support higher quality data exchange integrations within and outside of research. CDISC does not encourage the creation of standards, which are already well developed by other organizations; rather we seek to find ways to harmonize and connect our standards to these standards. With this principle in mind we asked EAB members what other data standards would be most important to consider collaborating/connecting with for this initiative and why. Here is a list of existing standards provided by respondents provided and their rationale for recommending them:

COMMON DATA ELEMENTS (CDEs)

- A structured item characterized by a stem and response options together with a history of usage that can be standardized for research purposes across studies conducted by and for NIH. NOTE: The mark up or tagging facilitates document indexing, search and retrieval, and provides standard conventions for insertion of codes. [NCI, CaBIG]. See also item, item (PRO), stem, data element, data element identifier. (CDISC Glossary, 2.0, downloaded from https://www.cancer.gov/research/resources/terminology/cdisc#cdisc-glossary)

REGISTRIES

- National Database for Autism Research (NDAR)
  - An NIH-funded data repository that aims to accelerate progress in autism spectrum disorder (ASD) research through data sharing, data harmonization, and the reporting of research results. NDAR is one of several databases that make up the National Institute of Mental Health Data Archive (NDA) (https://nda.nih.gov/about.html).

CLINICAL INFORMATION MODELS

- Clinical information models / archetypes (e.g., openEHR, openCEM, CEDAR)

HL7 FHIR

- https://www.hl7.org/fhir/

ICH

- ICH is not the council for data standards, but since they have recently discussed GCP renovation and protocol template, it may be important stakeholder in the context of utilization of RWD for regulatory purpose.
- The two major sources for real-world data (RWD) in medical research are Integrated Addendum to ICH E6(R1), ICSR E2B R2 and R3 and MedDRA

ISO

- IDMP standards defining product and registration data that is RWD (https://www.fda.gov/industry/fda-resources-data-standards/identification-medicinal-products-idmp)
- ISO13606, ContSys, HISA: EHR data

mCODE

- mCODE, as leveraged by ASCO CancerLinQ - I do not know a lot about this initiative, but they are collecting a ton of data - first for quality but now for research using their limited set of standards. The data come right from the HER (https://mcodeinitiative.org).

NATIONAL CANCER INSTITUTE

- NCI's Cancer Research Data Commons initiative - especially the Center for Cancer Data Harmonization (CCDH) - this group is studying a broad range of standards and helping data collection nodes with standards implementation (https://datascience.cancer.gov/data-commons).

IEEE - IEEE medical devices data standards that has RWD (https://www.ieee.org).

ICHOM - International Consortium for Health Outcomes Measures (https://www.ichom.org).

OMOP COMMON DATA MODEL by OHDSI (https://www.ohdsi.org/data-standardization/the-common-data-model/)

- OMOP and FHIR are important. OMOP already has a data model. OMOP also considers terminology invulnerability. I think the terminology is most important for data linkage.
- OHDSI use the standards, Observational Health Data Sciences and Informatics (OHDSI), (https://www.ohdsi.org/data-standardization/the-common-data-model/).
- OMOP: observational data
- OMOP which is used when working with healthcare data...just for awareness...many around the world are using this because it is easy to understand for healthcare uses and research using healthcare data (https://www.ohdsi.org/data-standardization/the-common-data-model/).

TERMINOLOGIES

- Expanding the integration of controlled vocabularies with CDISC, including standard drug names and MEDRA (<https://www.meddra.org>), would be very useful.
- National Cancer Institute, Enterprise Vocabulary Services (NCI-EVS) controlled terminology (https://evs.nci.nih.gov).
- Collaboration among other prevalent healthcare and research data standards and concept system developers, such as ICD (https://www.who.int/classifications/icd/en/), SNOMED (<http://www.snomed.org>), LOINC (https://loinc.org)., to harmonize where possible, and with software and Extract, Transfer, Load (ETL) tool vendors to agree on standardized machine readable formats for data standards so they could be published in these formats and used by software developers to make it easier for data users to aggregate and transform data that has been represented in one of these standards.
- SNOMED CT (SCT) is important. But you cannot use it without licenses. If CDISC is considering promoting the standards to multiple countries, including China, Japan, which are not yet SCT members, there must be a method to solve the licensing issue.
- Again, may not be standards exactly but terminology groups like SNOMED-CT, ICD 11, NICHD, NCI-EVS,
- MedDRA and SNOMED as they are widely used and are of importance to my area of research.
- MedDRA and WHO-DD (https://www.who-umc.org/whodrug/whodrug-portfolio/whodrug-global/). These are important because they are essential for standardization but expensive.
- Terminologies (SNOMED CT, LOINC).
- MedDRA and WHO-DD. These are important because they are essential for standardization but expensive.

HIPAA & GDPR

- HIPAA and GDPR are the regulation/law that must be taken into consideration (<https://www.hhs.gov/hipaa/index.html>) (https://gdpr-info.eu)

ONTOLOGIES

In the rare disease field, ontologies are very important, these are not exactly standards per se, but need to be factored in (e.g. Human Phenotype Ontologies (HPO), Orphanet Rare Disease ontology (ORDO).

PCORNET

- PCORNet (https://www.pcori.org)

SENTINEL

- Sentinel (https://www.fda.gov/safety/fdas-sentinel-initiative).

IMAGING

- Probably the area of imaging would need to be considered in depth, as so many medical procedures presently rely on imaging and the electronic health record data may only indicate the limited information that imaging was conducted.

GENETICS

- The clinical genetic standards needed is another large activity in my opinion.

USCORE

- ONC USCDI (US Core Data for Interoperability) which is setting data collection and implementation standards for EHRs. https://www.healthit.gov/isa/united-states-core-data-interoperability-uscdi

COMET Initiative

- COMET initiative (core outcome sets). “A core outcome set (COS) is an agreed standardised set of outcomes that should be measured and reported, as a minimum, in all clinical trials in specific areas of health or health care." <http://www.comet-initiative.org>

ARTICLES RECOMMENDED

- Use of HL7 FHIR as eSource to Pre-populate CDASH Case Report Forms using a CDISC ODM API. Sam Hume, CDISC, State College, PA, USA Jeff Abolafia, Rho, Chapel Hill, NC, USA Geoff Low, Medidata Solutions, London, UK. <https://pdfs.semanticscholar.org/1692/cf8e82fb9229baaba2eea03f0fc27f66aaa6.pdf>

GENERAL COMMENTS

- This is no silver bullet or single standard that can address interoperability, but we should streamline standards and reuse and enhance what is available. FHIR will not fix or address everything but it is an exchange standard that healthcare organizations will use and should be considered for research. Why create and map?

SUGGESTED COLLABORATIONS

- The Office of the National Coordinator is involved with regulations around standards use to increase quality and improve clinician experience with EHRs. Most of their work is around FHIR. https://www.healthit.gov
- Work with the CTSA which are academic centers conducting research across the nation. (NCATS). https://ncats.nih.gov/ctsa
- Examples include CD2H and sIRB project. https://clic-ctsa.org/publications/ctsa-program-national-center-data-health-cd2h
- There are standards for RWD. Learning how best to leverage, enhance and build on them is the next step.
- CONSORT for EHEALTH - may provide relevant pointers on evaluation of digital health interventions. https://www.jmir.org/2011/4/e126/

## The Future of RWD and CDISC Standards

Finally, we asked the EAB about their vision for the future. First, we asked them to forget for a moment about time and resource limitations, what would be three wishes that could be fulfilled to make implementing CDISC standards for RWD easier. Their replies were the following, with 78% strongly agreeing with them, and the rest moderately agreeing:

**Simple and flexible templates and tools (28)**

- Development of educational tools for beginners.
- Master user guide of mapping of data, data dictionary.
- Easy to use data collection tool with inbuilt CDISC standards.
- Promote use of global open source file formats.
- Better implementation tools.
- CDISC RWD for dummies i.e. intuitive guide on end to end implementation, best practice, validation guide.
- Open access tools.
- Make the tools available to all to access.
- 'Meaningful use' of existing standards, which would translate to true interoperability.
- Provide a free system that implements the CDISC standards as a globally unified system.
- Annotated CRFs for every medical fields, including any RWD sources.
- Implementation guide for every medical fields, including any RWD sources.
- Effective system that prevent incorrect implementation of CDISC standards.
- Library of recommended and validated metrics using RWD.
- Translation of CDISC documents into Chinese and other languages.
- Low cost for implementing CDISC standards.
- Data transformation from events/encounters to a study-oriented data model.
- If 'easier' means 'more correct', then: think thoroughly about the meaning of the data being modelled, construct models that capture those meanings.
- Software engineering artifacts that streamline implementation of the standards in software.
- Healthcare and clinical and researcher information systems that have already implemented the standards, so researchers do not have to worry about it.
- Know all the pitfalls ahead of time.
- More accessible, easier to understand.
- Plug-and-play tooling.
- Seamless integration with other standards for modeling (ISO & OMOP, among others).
- NO Mapping!!!
- Harmonizing the standards.
- Development of open-source tools for metadata validation and mapping to CDISC standards.
- Easy to find, easy to follow and readily automated procedures to implement CDISC standards.
- All of the FDA Therapeutic Area standard data elements should be implemented as CDASH data elements.
- Data elements should be created for all major TAs.
- CDASH data elements should be mapped (in computationally useful format) to FHIR.
- Point and click interface that creates CRF inclusive of RWD.

**Free or affordable training and education (15)**

- Training materials to get academic researchers using standards.
- Providing training focused on academic use of the CDISC standards, providing templates and mapping tools, providing portal site for information sharing.
- Hold many national and international conferences to increase public awareness of standards for RWD and provide training to the stakeholders on what is RWD and the utility of RWD.
- A retreat with one-on-one training on how to a) create a new resource e.g., an app or a registry based on CDISC standards AND b) how to evolve an existing resource to run on CDISC standards. This would be expensive perhaps as would need to be bespoke: maybe a BYOD (bring your own data) sort of event.
- Education for medical professionals.
- Free on-site training/online and education for AMCs AND sponsors.
- Human resource development for CDISC experts in academic institutions.
- Education for patient.
- Clear understanding of rationale, scope, and inner workings of CDISC standards.
- Free training on using the standards.
- Educational activities and working with research community and regulators.

**Support standardization of EHR data (12)**

- Less open text fields in EHRs.
- AI extracts data from Doctor notes and puts in standard format.
- Use of Artificial Intelligence (AI) data on EHR is automatically converted to CDISC standards format, readily available CDISC compatible CFR creation, converter of local language to CDISC standards map.
- All electronic health data from all sources in CDISC format.
- Using new technologies, including NLP, to convert the EMR data to a database following CDISC standards in multiple institutes and countries.
- Involve vendors and design a system that uses the CDISC standard from the electronic medical record and RWD data sources and upstream of the data.
- All laboratory data from all sources in CDISC format.
- Incentives for EHRs vendors will be needed.
- Healthcare and clinical and researcher information systems that have already implemented the standards, so researchers do not have to worry about it.
- CDISC (CDASH) should focus on research specific content rather than healthcare (e.g. protocol, PI, experimental treatment, IRB) which is already standardized in many situations. OR work closely with healthcare to align efforts.
- Integrate with existing healthcare standards so we are using fewer standards (single data collection standards, single format standards, single exchange standard) for various uses.
- CDISC to collaborate closer with HL7.
- HL7 to collaborate closer with CDISC.

**Use cases (8)**

- Use cases.
- Good examples.
- Develop a lot of use cases and data.
- A large-scale study addressing a disease area or a specific medication and support better decision making or repositioning of the medication.
- Clearly summarized and explained use cases of how different academics and different resources have embraced CDISC standards and what this has enabled them to do which could not be done before
- Some working examples.
- CDISC should promote use cases in clinical research by close collaboration with academia, EDC and EHR vendors and biopharma companies.
- Making CDISC-formatted example data publicly available

**Standardize terminologies (7)**

- Standardization of different terminologies.
- Bridging the terminology mismatches, most notably between MedDRA and ICD codes.
- Constraining LOINC codes for labs (or having a new and better standard for laboratory data available)
- Seamless integration with other standards for terminology.
- Work with controlled terminologies in use by Healthcare, (ICD-10, SNOMED CT, LOINC, etc.).
- Extended scope of terminology development (to support population-based, epidemiologic, public health, and translational research including the genomic aspects.

**Simplification (7)**

- Minimize the number of standards; strongly support the use of available global data standards or the development of new standards in fields where none are available to ensure early alignment.
- I would support the use of available data standards only if those SDOs are willing to make their data standards better. The reason that RWD and healthcare data are so messy is because the existing standards are simply not that good.
- Much more efficiency for downstream data analysis after data standardized.
- Think the specification should be simple.
- Simplicity, broad and modular to accommodate many different study types.
- Make it easy to implement the standards, with flexible usage.
- We need to focus on important content and preserve connections so if we need, we can get more data later.
- Design these standards based on what is the primary endpoint. Develop granular data elements to derive the primary endpoint. Once this is done, these elements can be required in any standards development effort and data pipelines can be created to get exactly what we need.
- Simplification is only possible insofar as how ‘simple’ the data is. In real life, data is complex, and the standard should be able to meet that complexity. The downside of ‘simplifying’ is the incorrect structuring of data.

**Regulation and requirements (6)**

- Regulation.
- Where data cannot be standardized at inception, establish the regulatory requirements to confirm the validity of mapped data.
- Journals requiring CDISC standards for publication datasets.

**Ongoing support for implementation (5)**

- A large professional IT team is needed.
- 24-hour support.
- Analytic support.
- Available data standards experts and data warehouse technicians to help with standards implementation on site.
- One-on-one support.

**Champions (5)**

- A high-level policy incitement to use of CDISC standards - either from an influential country in its health system, for deployment of EHRs; or a strong statement from the EMA for instance.
- A far-sighted leader who masters the information resources should know about the enormous value of using CDISC standards.
- Private-public partnerships.
- Inclusion of stakeholders in low- and middle-income countries.
- Cooperation among sponsors and among academic institutions.

**Well-defined purpose and scope of CDISC standards for RWD (4)**

- The purpose and scope of implementing CDISC standards should be overall assessed.
- Define the issue better.
- Confidence that the CDISC standards really are the optimal way to collect and store RWD.

**Financial support (4)**

- Financial support.
- Enough budget to support standards for RWD, enough human resources to work on standards for RWD.
- Academic funders jointly supporting the adoption of the standards for all research they fund.
- Support to design a multi-disease, multi-domain Study Data Tabulation Model (SDTM) data repository.
- Create the pull from funders.
- Academic funders jointly supporting the adoption of the standards for all research they fund.
- Harmonization among funders' requirements.

**Other**

- Standardized data collections
- Clear method of alignment between UK Hospital Episode Statistics (HES) data and SDTM. https://digital.nhs.uk/data-and-information/data-tools-and-services/data-services/hospital-episode-statistics
- CDISC should be run more openly.

**RWD Standardization in 3-5 years**

One of the key objectives of this project was to understand what a future of CDISC standards and RWD might look like three to five years in the future. To this end, we asked participants what they hoped to be able to do in three to five years with regards to RWD standardization and utilization. The majority wanted to be able to share or aggregate routine healthcare data and to use RWD in regulatory submission and public heath purposes. Others responded that sharing and aggregating patient level registry data and standardizing multiple sources of RWD to consolidate and generate meta-analysis was desirable.

|  | **High Priority** | **Priority** | **Not a Priority** |
| --- | --- | --- | --- |
| Standardize multiple sources of RWD to consolidate them for other purposes | 8 | 21 | 11 |
| Standardize multiple sources of RWD to generate meta-analysis | 13 | 22 | 9 |
| Share or aggregate patient level data from registries | 15 | 20 | 9 |
| Standardize RWD for public health purposes | 19 | 17 | 8 |
| Utilize RWD as inputs for regulatory submissions | 22 | 10 | 12 |
| Share or aggregate routine healthcare data | 26 | 11 | 7 |

**Other future state priorities:**

Responses related to a desired future state of

- Improve data validation and data quality.
- Aim to provide a multi-language system of standards from the beginning. Cover different important markets, especially in Asia.
- Integration of research and non-research data based on elaborated standards of data quality assessment and integration standard.
- Arrange SDTM specs to adapt RWD characteristics, especially “Highly Recommended” versus “Required” core designations in SDTM rules.
- Collaborate with and disseminate information with organizations that promote information disclosure.
- Collect once use for other stakeholders, digital health quality data aggregation, decreasing research workload clinical setting.

**Areas of Non-agreement**

- Diagram of RWD - the definitions of the different components of RWD are not agreed upon by participants
- Focus on EHR first - some outlier participants disagreed, and some participants stated implementation would be difficult
- Registries - some said they should be a priority and others said they should not be a priority
- Make CDISC standards easier to use before attempting to expand, versus improving standards in parallel with exploring and testing the expansion of use with RWD.

# Use Cases

## IDDO Use case – Novel SDTM implementation to Improve Legacy Data Sharing

Launched in 2016, and born out of the success of the WorldWide Antimalarial Resistance Network (WWARN), the Infectious Diseases Data Observatory (IDDO) is a scientifically independent, multi-disciplinary coalition of the global infectious disease community, with WWARN as the malaria division of IDDO. It provides the methods, governance and infrastructure to translate data into evidence that improves outcomes for patients worldwide.

IDDO is working on a variety of active and planned research themes that call for the consolidation of data from multiple sources, including:


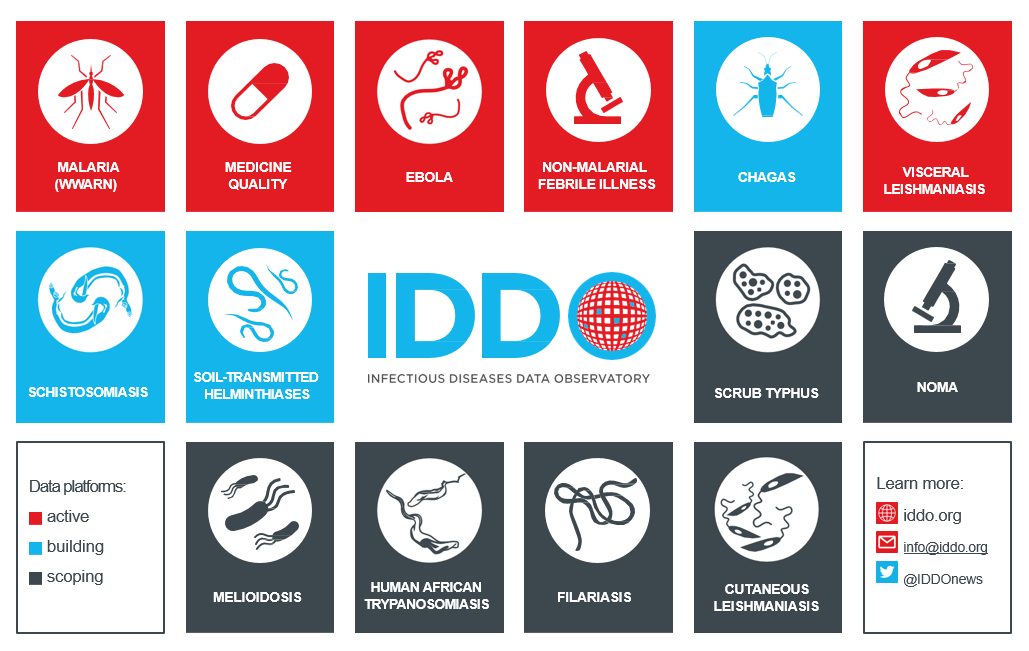


The creation of each of IDDO’s pathogen-focused data repository begins with the development of a research agenda, which is collaboratively developed by IDDO, researchers and other stakeholders (for an example of the Ebola research agenda with prioritized research questions, please visit: <https://www.iddo.org/document/ebola-research-agenda-public-consultation-plateforme-de-donnees-ebola-programme-de>). Afterward, the group conducts a systematic review of the published data available to identify relevant research and public health data, and also contacts disease experts about eventual unpublished data from grey literature, such as an NGO’s study report of their trials. The groups inquires specifically about contributions of data that would help answer the questions on the research agenda. Accepting contributors provide individual patient data in any format, and data are cleaned, standardized, and mapped by a curator. Finally, data are added to the IDDO data repository in standardized format using SDTM. The integration of hundreds of standardized datasets allows IDDO and the research community to reuse the data for multiple meta-analysis that generate new results with strong statistical power thanks to the large number of data points. The results of the meta-analysis are published in peer-reviewed journals, and they are used to inform policymaking and the development of treatment guidelines.

The IDDO platform includes legacy data from clinical trials, outbreak responses, longitudinal studies, public health programs, pharmacokinetic studies and genomic studies conducted by external researchers. The platform accepts any individual patient health data that can support analyses to address the platform-specific research agendas. The IDDO repository includes the following types of variables:

• Testing data

• Treatment data

• Signs and symptoms data

• Patient information

- Pathogen genotype data
- Pharmacokinetic and pharmacodynamic data
- Epidemiological data

• Outcomes

IDDO has leveraged the accessibility, user friendliness and reproducibility of the SDTM standard to create a specialist IDDO CDISC Data Dictionary with a built-in implementation guide, specifically created for the aggregation of data for reuse. The Dictionary is disease-agnostic and covers a wide range of data types, allowing diverse data to be pooled for reuse in meta-analysis. To date, SOPs for the data curation process as well as the initial draft of the IDDO CDISC Data Dictionary have been created and are being trialed with data from five disease areas. Completion rules, examples for application to disease-specific variables, and applicable controlled terminology are continually added as new data is shared and curated. With the addition of new data, new diseases and new data types, the Dictionary is iteratively expanding and developing. This approach has enabled the creation of a single IDDO Data Repository, a platform with the end goal of reducing the impact of poverty-related infections by generating new evidence through data reuse.

This is easier said than done. Building a disease-agnostic CDISC data dictionary has been challenging because a multi-study, multi-disease application of the standard may require more flexibility where rigidity exists – and vice versa. The SDTM implementation guide is designed for application to a single study. Certain definitions and rules have a vagueness which can be clearly applied to a single study, but becomes problematic when several people are applying a rule to many studies that will be grouped together. Also, some of the rules and definitions are quite restrictive or complex in order to follow the SDTM implementation guide for regulatory admissions; following some of these did not make sense for IDDO’s use case. It was additionally complex due to the distribution of resources, controlled terminology, and examples across a number of locations. These all needed to be collated into a single cohesive document to enable curation.

Some types of variables are pretty straightforward to map to SDTM, including vital signs and functional clinical test results, as well as testing and lab data such as hematology, biochemistry, and microbiology. Other variables, including treatment data, are harder, especially when it is not trial data, when there are atypical treatments conducted, and when cross dataset comparisons need to be performed.

In order to future proof their application of SDTM, IDDO has adapted the standard to fit their needs with future analyses always in mind. The development of the Dictionary was based on an initial package of distinct diseases and data to avoid creating rules based on a single dataset and disease, which would not accommodate the diversity of data bound to be found in future datasets. Applications of the rules were therefore designed to accommodate the variety of data of interest to the IDDO community. It is not possible to know in advance all the types of analyses that data could be reused for. Therefore, decisions were made with the end-user in mind, to ensure that as many data as possible were captured in the most useful and user-friendly format.

The main lesson learned from the implementation of SDTM for real-world data (RWD) is that standardization needs to be balanced with flexibility. Repositories of RWD need to be clear, concise, and accessible for everyone, while also remaining comprehensive, flexible and dynamic. The IDDO experience has shown that SDTM is sufficiently flexible to accommodate the variety and integration of data required to address major challenges in poverty-related infections. However, certain challenges to fit the RWD into the standard have resulted in some rule bending, and since some parts of SDTM may not apply to the data in the repository, IDDO may have to work with data ‘as is’. Finding a way to combine rigor with creativity has been a key component to IDDO’s success. https://www.iddo.org

## Finger Lakes Use Case - Application of SDTM to the NY State Perinatal Data System

Frontier Science is a not-for-profit organization founded in 1975, focused on innovative data management and statistics, collaborating with research networks, pharmaceutical companies and others in the design, conduct and execution of clinical trials and long-term observation studies.

Some Frontier Science’s partners currently have studies that gather data on pregnancy and birth outcomes. Noticing that some of the data in a New York State perinatal and health outcomes registry mirrored the outcome forms in their clinical trials, and given that pregnancy data is not collected enough in research studies and is very much needed to address health issues during this critical human developmental stage, Frontier Science decided to test the implementation of the SDTM standard on the registry. The Finger Lakes Birth Records registry includes births within 9 counties in New York State (Monroe, Wayne, Livingston, Ontario, Yates, Seneca, Steuben, Schuyler, and Chemung), which cover a mix of urban and rural population and a total of over 1.2 million people. Between the years 2011 and 2015, there have been over 68,000 birth records included.

Since Frontier Science did not have access to the full dataset, they worked with the registry tables with the data stripped out, looking at how the data was stored and how it could mirror the SDTM format. The source form is composed of 9 pages, 408 fields, and they contain a mix of patient responses and responses from different medical personnel involved in the birth. There are 193 rows of data per labor event to be input into SDTM, including 14 domains, as follows:

- AE- Maternal morbidity information
- CE- Congenital anomalies, low birth weight delivery information
- DM- Newborn demographics
- FA- Plurality and birth order, birth history, start of prenatal care
- MB- Syphilis serologic test
- MH- Prior pregnancy outcomes, infertility treatments, risk factors
- PE- Newborn screening
- PR- Fetal genetic testing, labor and delivery information
- QS- Medical interview with the mother
- RP- Menses information
- RS- APGAR scores
- VS- Maternal and infant vital signs
- SU- Alcohol, tobacco, and substance use
- APDM- Maternal and second parent demographic information

**Challenges and lessons learned from the use case**

Several questions surfaced during the implementation of SDTM. Regarding pregnancy-related data, the team found the challenge of how to deal with multiple persons being the focus of the study and who should be considered associated persons (e.g. fathers, medical providers, etc), whether AP domains were appropriate, or whether there was a need to use a flag for mother, infant, attending, etc. Another question that came up was whether the source of the information (patient or provider as the source) should be differentiated in SDTM, and how. Finally, it was not clear by the team how prenatal risk factors should be handled in the model, and whether the birth details should be a domain or a suite of domains.

With regards to public health-related data, there were several technical aspects that surfaced, including variables that are required in SDTM such as subject identifiers and the STUDYID variable, which do not exist in a public health setting. Of note, the registry is nominal for each birth, and the mother could also be potentially identified depending on the data that is made available. Related to this, the team also realized there is insufficient guidance on how to handle Personally Identifiable Information (PII) and HIPAA regulations in the implementation of SDTM. Informed consent was also an issue identified by Frontier Science, since in this database there is a number of people in addition to the mother (including the partner and other health practitioners) providing data but presumably not all of them have given their informed consent. Even in the case of the mother, who usually has given her written consent to receive care and have data collected, it is not clear what exactly they are consenting to. Additionally, a discussion is needed around the potential use of standardized data across multiple countries, in light of new regional efforts such as the European Union’s General Data Protection Regulation (GDPR) on data protection and privacy.

The team also wondered what needs to occur with CDISC Controlled Terminology to accommodate public health research and other academic work outside of regulatory submissions. Looking at the types of medical providers, for instance, there are some terms that do not exist, like a doula. Also, different types of cleft palates are not part of controlled terminology, and defining whether things happen prenatally, perinatally, or postnatally was also absent.

As next steps, Frontier Science recommends having conversations about handling both these types of data: public health and pregnancy data, with the latter being of high priority for research and public health beyond regulated trials. The conclusion of the team after the use case is that SDTM works with pregnancy data, but guidance on how to store it in a cleaner way needs to be developed. Additionally, significant changes are needed in the public health and academic setting for the adoption of SDTM and the CDISC standards. There are components of data standardization that are taken for granted in clinical trials, such as selection of participants, data sources and data quality, flexibility with adopting new technologies, which are much more complex in academic and public health activities. The team recommends discussing how people think about data storage, data quality, data cleanliness, where the CDISC standards come in to disrupt the status quo, and how to make it more appealing for academics and public health people to adopt them.

## PAHO HEARTS Use Case – Mapping of Hypertension Program Data into CDASH

HEARTS is an initiative spearheaded by the World Health Organization involving various global actors: including the Pan American Health Organization (PAHO) the Centers for Disease Control and prevention of the United States (CDC) and the Initiative Resolve to Save Lives, the International Society of Hypertension and Nephrology, the World Hypertension League and World Federations of Diabetes and Heart. The HEARTS technical package supports Ministries of Health to strengthen cardiovascular disease management in primary health care settings. HEARTS in the Americas is an initiative of the countries, led by the Ministries of Health with the participation of local actors with the technical cooperation of PAHO/WHO, which seeks to integrate smoothly and progressively to already existing health delivery services to promote the adoption of global best practices in the prevention and control of cardiovascular diseases (CVD), and improve the performance of the services through better control of high blood pressure and the promotion of secondary prevention with emphasis on the primary health care.

CDISC collaborated with PAHO/WHO to conduct a mapping of the countries’ reported data points into CDASH. Additionally, CDISC created visualizations and metadata table examples for PAHO to adapt and to promote with HEARTS-participating countries, with the aim to receive national-level data with greater standardization. The CRF to be created will include standard questions, standard definitions, and standard options of answers.


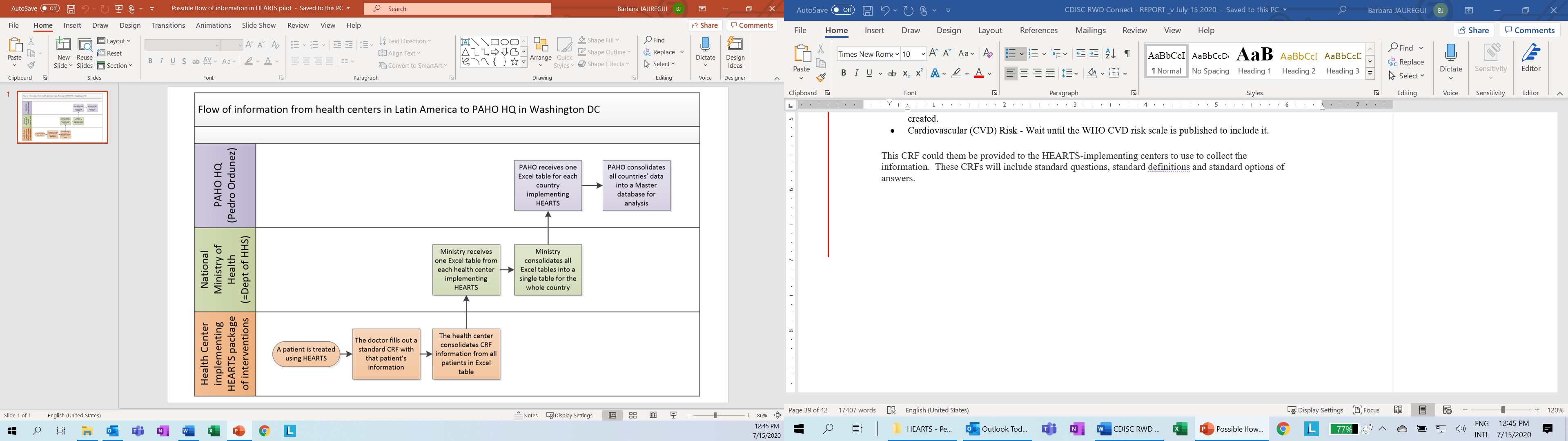


CDISC reviewed PAHO clinical variables and performed a gap analysis against core CDASH/SDTM domains and variables as follows:

- Socio-demographics
  - age
  - sex
  - SES
- Subject characteristics
- Vital signs
  - blood pressure measurements
- Medical history
  - other diagnosis
- Concomitant medications
  - blood pressure medications

Two of the concepts included in the data collected needed additional work, while the remainder of the concepts being readily mapped into existing standards. There were however, two concepts that required work. These were:

- - - - *Socioeconomic Status* - there is currently no CDISC controlled terminology for a test of "Socioeconomic Status". PAHO then suggested terms that are commonly used in the LAC Region ("low", "middle", "high" and their definitions), and CDISC requested the terminology to be created.
      - *Cardiovascular (CVD) Risk* - the WHO CVD risk scale needed revision and CDISC had to wait for it to be published.

From this initial review of PAHO public health data points the CDISC standards easily accommodated routine public health data.

## Clinical Innovation Network (CIN) Use Case – Gap Analysis between Remudy and TAUG-DMD (and related the other CDISC Standards) to Construct Patient Registry for International Data Sharing

**Introduction**

In 2007, TREAT-NMD which is the research network for neuromuscular disorders, was established in European country collaborate with united states and the other lesion. This network had many activities, and one of the most important issue was global registry. They planned patient registries as a tool for research, trial readiness, natural history and others, and were willing to integrate each registry to global registry.

In 2009, following TREAT-NMD, Japanese muscular dystrophy group launched Nationwide registry of DMD/BMD patient collaboration with TREAT-NMD, the Registry of Muscular Dystrophy (Remudy) at National Center of Neurology and Psychiatry (NCNP). The purpose of this registry was to “effectively recruit eligible patients” to new clinical trials and “provide timely information” to patients about upcoming trials. Registry data also provides more detailed knowledge about the “natural history” and “epidemiology” of the disease, as well as information about clinical care. As of the end of December 2019, the number of registrants at the Remudy has 1,905 dystrophinopathy, 215 DMRV (GNE myopathy), 959 myotonic dystrophy, and 54 congenital muscular diseases (http://www.remudy.jp/news/2020/01/005944.html). In 2016, the disease registration system (Patient registry) for clinical development, the clinical innovation network (CIN) concept aiming to revitalize domestic clinical development. The other one is some researches using new data such as RWD on the regulatory use on the issues for harmonization of international regulations are carried. In order to promote the utilization of RWD, it is important to ensure data reliability and data traceability from data collection to data exchange, analysis and storage of data. Standardization is necessary for that.

CDISC provides consistent standards from planning, data collection, data tabulation, and analysis, and is useful for ensuring data traceability. In order to use registry data, how to collect the data and how to ensure the quality of the data would be a problem, so we thought that using the CDISC standards would help to solve the problem.


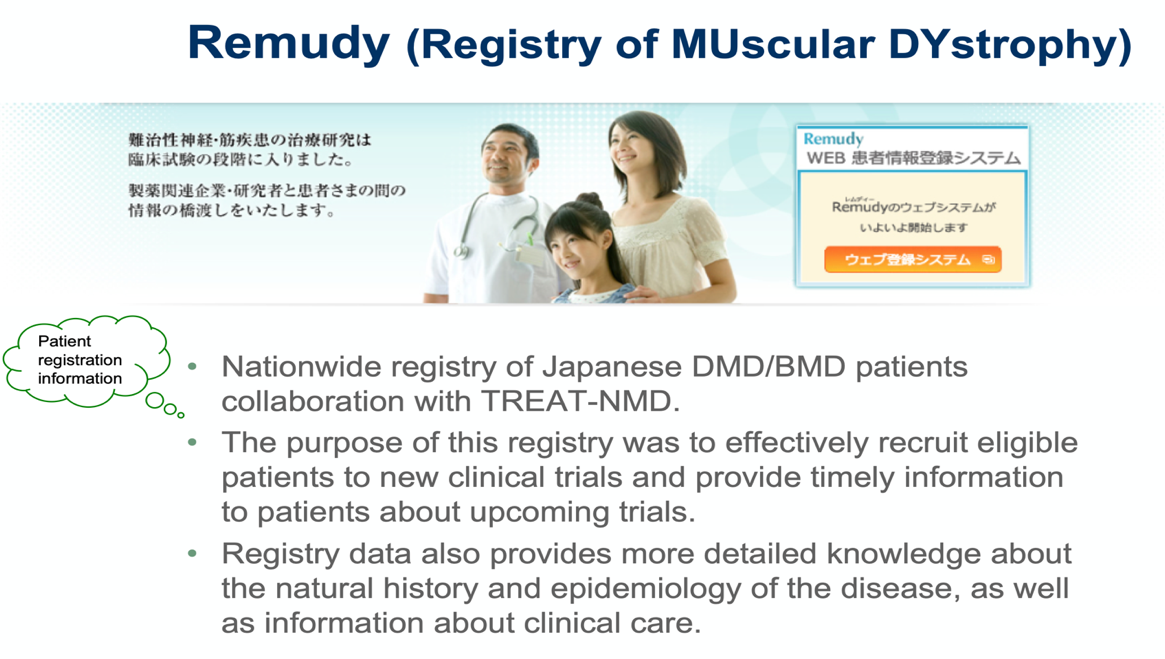


**Objectives**

The collection items of Remudy collaborate with TREAT-NMD, but it is important that data can be used internationally. Because TAUG-DMD follows one of the CDISC standards, it was very important information to examine whether the collection items of the existing DMD patient registry were internationally valid and to compare them. This is the good opportunity to refer the international standard. The Duchenne Muscular Dystrophy Therapeutic Area User Guide (TAUG-DMD) version 1.0 was developed under the CFAST Program, and CDISC released TAUG-DMD v.1.0 on 25 September 2017 (https://www.cdisc.org/standards/therapeutic-areas/duchenne-muscular-dystrophy).

We conducted to gap analysis on the collection items and the responses of DMD patient registry in Remudy (includes collections of planned natural history studies) and these of TAUG-DMD including related CDISC Standards. As a future goal of this research was below.

- To confirm the validity of TAUG-DMD and to examine its availability in current situation of Japan
- To develop a database of registries of DMD and the neuromuscular disorders
- To be one of the examples of building a patient registry using CDISC Standards

**Method**

The items collected by Remudy were compared in the order of the table of contents of TAUG-DMD. Remudy includes the following type of variables:

• Patient information

• Genetics data

• Disease assessments data

Assistive Devices data

Loss of ambulation data

Pulmonary Function Assessments data

Questionnaires, Ratings, and Scales

Ex. 6-Minite Walking Test (6MWT) (planned natural history studies)

• Assisted Ventilation data

**Registry standards were reviewed**:

- Nationwide registry of Japanese DMD/BMD Registry of Muscular Dystrophy (REMUDY)
- CDISC Standards
  - TAUG-DMD
  - Related the other CDISC Standards
- Standards related Rare Diseases
  - National Institutes of Health (NIH), Global Rare Diseases Registry Data Repository (GRDR)
  - European Union Committee of Experts on Rare Diseases (EUCERD)


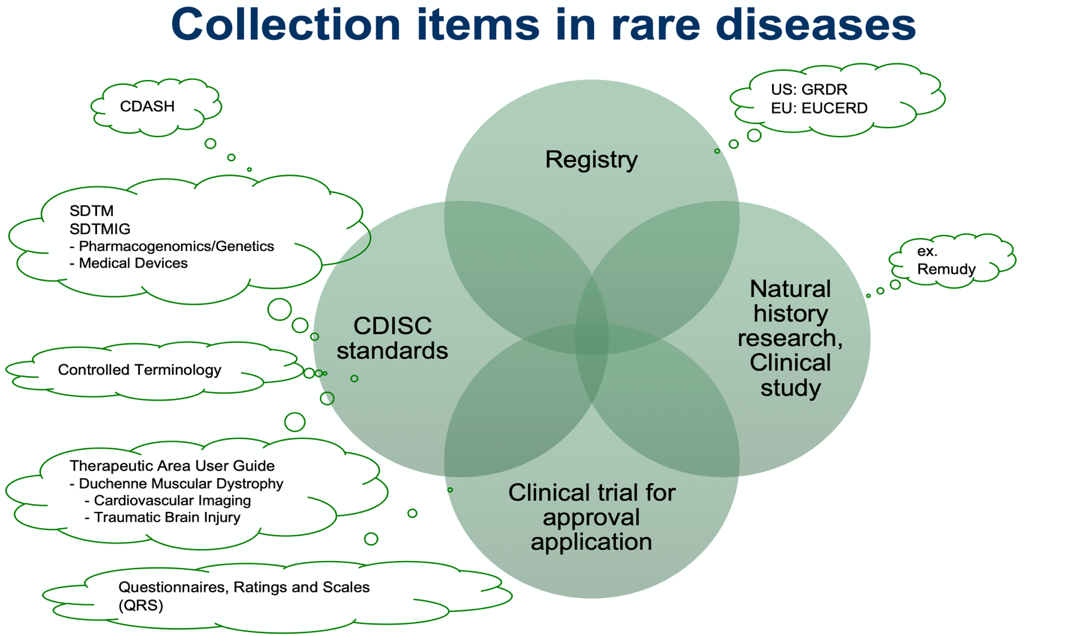


**Process**

- Compared collection items and responses based on Remudy and TAUG-DMD v1.0
- Examined the collection items considering CDASH from TAUG-DMD v1.0
- Compared the differences between collection items and responses considering the CDASH (TAUG-DMD) in Japan (ex. Remudy).
  - Genetic information (Genetics data)
  - Clinical information (Disease assessments data and Assisted Ventilation data)
  - Patient registration information (Patient information)

**Findings**

TAUG-DMD covered various necessary information for setting collection items and responses on the construction of the DMD patient registry.

In genetics information, the expression was unified in basic research and the information required in clinical practice was clear, suggesting that the test results were satisfactory. In clinical information, evaluation items were covered, but there were some differences in collected data. It was necessary to clarify the differences in the measurement method and measurement environment, and to enable more international data utilization. In the patient registration information, since there is no CDISC standard, it was necessary to check standards such as GRDR and EUCERD and confirm the collection items.

**Recommendations**

TAUG-DMD was a very useful resource in comparing the collection items of DMD patient registries. Because it was necessary to organize the information in PDF again in a table, it was useful to provide CRF and TAUG-DMD’s CDASH. Patient registries have the potential to collect persistent data and differ in the notion of study initiation and termination as in existing CDISC standards. For example, if respiratory function declines after enrollment, it may be considered a complication rather than an adverse event. The use of CDISC standards in patient registries requires proper interpretation of the CDISC standards in interventional and observational studies and requires a proper understanding of CDISC standards.

Considering the quality of data collection, it is advantageous to be able to understand the collection items and answers in each native language. Providing information in multiple languages ​​in addition to English may facilitate the implementation of the CDISC standard outside English-speaking countries. Also, considering international utilization of data, it is necessary to design the database at the same time. For this purpose, it is desired to provide a tool that can search the CDISC standards such as Controlled Terminology and related standards in addition to TAUG-DMD, SDTM, and CDASH, as well as the variable names and variables.

Although the collection items were the same, it was confirmed from the TAUG-DMD information that the measurement method was different. The difference in the measurement method was shared with CDISC by presenting it at CDISC Interchange, etc., and the more information sent from researchers, the more use cases will be increased, which will be useful for implementation. It is important that information exchange and information sharing with CDISC be carried out more actively. In addition, the patient registry is constructed and operated mainly by hospitals and medical institutions. To use the CDISC standard, there is a duty to pay a membership fee, but reducing the burden on medical institutions, such as the membership of medical institutions and exemption from annual fees, will lead to the spread of CDISC standards. In order to use medical data as research data, an environment that is easy for medical institutions to implement is required.

# References

*References used to inform the creation of the diagram:*

- US Food and Drug Administration. (2019). Submitting Documents Using Real-World Data and Real-World Evidence to FDA for Drugs and Biologics: Guidance for Industry: Draft Guidance. Rockville, MD: *US Food and Drug Administration.*
- Berger, M. L., Sox, H., Willke, R. J., Brixner, D. L., Eichler, H. G., Goettsch, W., ... & Wang, S. V. (2017). Good practices for real‐world data studies of treatment and/or comparative effectiveness: recommendations from the joint ISPOR‐ISPE Special Task Force on real‐world evidence in health care decision making. *Pharmacoepidemiology and drug safety*, 26(9), 1033-1039.
- Greenfield, S. (2017). Making real-world evidence more useful for decision making. *Value in Health*, 20(8), 1023-1024.
- Sherman, R. E., Anderson, S. A., Dal Pan, G. J., Gray, G. W., Gross, T., Hunter, N. L., ... & Shuren, J. (2016). Real-world evidence—what is it and what can it tell us. *N Engl J Med*, 375(23), 2293-2297.
- Mahajan, R. (2015). Real world data: additional source for making clinical decisions. *International Journal of Applied and Basic Medical Research*, 5(2).

# Appendix A – List of EAB Members

| **Name** | **Affiliation** |
| --- | --- |
| Yuki Ando | PMDA |
| Yoshihiro Aoyagi | National Cancer Center Hospital East |
| Adam Asare | Director of IT for the UCSF Breast Care Clinic |
| Wenjun Bao | SAS Institute |
| Lisa Blackwell | Oxford University |
| Greg Botwin | Cedars-Sinai Medical Center, Los Angeles |
| Francesca Cerreta | European Medicines Agency |
| Ronald Cornet | AMC UVA Netherlands |
| Jacqueline Corrigan-Curay | FDA-CDER |
| Emilie Darcillon | Nestle |
| Susana Dodd | Liverpool University |
| Janel Fedler | Clinical Trials Statistical and Data Management Center (CTSDMC), University of Iowa |
| Matt Fisher | Clinical Trials Statistical and Data Management Center (CTSDMC), University of Iowa |
| Jason Gerson | PCORI |
| Gideon (Scott) Gordon | FDA-CDER-OSP |
| Lorenzo Guizzaro | European Medicines Agency |
| Karin Hedenmalm | European Medicines Agency |
| Victoria Hedley | University of Newcastle |
| Ralf Herold | EMA |
| Shiro Hinotsu | Professor at the Department of Biostatistics at Sapporo Medical University, Sapporo University. Japan ARO Council. |
| [Ingeborg Holt](mailto:Ingeborg.Holt@ibm.com) | IBM, Global Business Services |
| Lynn Hudson | The Critical Path Institute |
| Trevis Huff | Clinical Trials Statistical and Data Management Center (CTSDMC), University of Iowa |
| Georgina Humphries | The Wellcome Trust |
| Virginia Hussong | FDA-CBER |
| Masakatsu Imoto | AMED Japan |
| Scott Kahn | The Harry B. and Lenora Helmsley Foundation |
| Matilde Kam | FDA |
| Qingna Li | Xiyuan Hospital, China Academy of Chinese Medical Sciences |
| Daisuke Koide | University of Tokyo, member of National University hospital council ARO TG3 |
| Sang Gyu Kwak | Daegu University Medical School, South Korea |
| Junkai Lai | Peking University Clinical Research Institute |
| Hanneke Lankheet | Danone |
| Becca Leary | University of Newcastle |
| Eri Matsuki | AMED |
| Carolina Mendoza-Puccini | NIH-NINDS |
| Gong Mengchun | Management Board of SNOMED International, Fudan University Children's Hospital, Peking Union Medical College Hospital. |
| Laura Merson | Infectious Diseases Data Observatory (IDDO), Oxford University |
| Erin Muhlbradt | NIH-NCI-EVS |
| Marianne Munene | KEMRI - Wellcome Trust Kenya |
| Lillian Mwango | KEMRI - Wellcome Trust Kenya |
| Harumasa Nakamura | Institute of Genetic Medicine, Newcastle University, Newcastle upon Tyne, UK. National Center of Neurology and Psychiatry, Tokyo, Japan |
| Mihoko Okada | Institute of Health Data Infrastructure for All (IDIAL) |
| Frank Pétavy | European Medicines Agency |
| Vaishali Popat | FDA-CDER |
| Kenneth Quinto | FDA- CDER |
| Christina Reith | Oxford University |
| Lyubov Remennik | NIH-NCI-EVS |
| Mitra Rocca | FDA-OCS |
| Frank Rockhold | Duke Clinical Research Institute; Duke University |
| Yaoping Ruan | LinkDoc technology, Beijing |
| Toshiki Saito | NHO Nagoya Medical Center |
| Maria Shin | Daegu Catholic University Medical Center, South Korea |
| Erin Sizemore | Centers for Disease Control |
| Enti Spata | Oxford University |
| Will Stevens | Oxford University |
| Catrin Tudur-Smith | Liverpool University |
| Satoshi Ueno | National Institute of Public Health Japan |
| Sam Volchenboum | University of Chicago |
| Naomi Waithira | MORU, Bangkok, Thailand |
| Anita Walden | University of Arkansas, Medical Sciences, HL7 |
| Jason Walker | Harvard Medical School, Massachusetts General Hospital |
| Karl Wallendszus | Oxford University |
| Denise Warzel | NIH-NCI |
| Jon Yankey | Clinical Trials Statistical and Data Management Center (CTSDMC), University of Iowa |
| Chen Yao | Peking University Clinical Research Institute |
| Meredith Zosus | University of Arkansas, Medical Sciences, or UT Health Sciences Center, Houston, HL7 |

# Appendix B – Survey Questions

SECTION 1 - Questions for background information

This section is intended to provide a better understanding of the participants' knowledge and experience in different areas relevant to the CDISC RWD Connect Initiative. Your individual responses will be seen exclusively by the organizers. We will only provide consolidated anonymized information back to the Advisory Board members.

1. First and last names of the people who contributed to the answers provided to this survey
2. Relevant institution/s you are affiliated with (mark all that apply and specify the name of the institution)

- University
- Research center
- Non-profit organization
- Government organization
- International organization
- Other:

**Your experience with Real-World Data (RWD)**

While there are multiple definitions of real-world data (RWD) currently in use, the CDISC Glossary has adopted the following definition, which is aligned with the FDA's definition: “*Data relating to patient health status and/or the delivery of health care routinely collected from sources other than traditional clinical trials. Examples of sources include data derived from electronic health records (EHRs); medical claims and billing data; data from product and disease registries; patient-generated data, including from in-home-use settings; and data gathered from other sources that can inform on health status, such as mobile devices.”*

With this definition in mind, this section next is focused on capturing your experience using RWD.

3. What is your experience using real-world data (RWD)? (mark all that apply)

- I have conducted experimental research/academic studies using RWD that were not intended for regulatory submission.
- I have conducted observational research studies (cohort study, case-control, etc.).
- I have worked with multiple RWD sources to conduct research around healthcare delivery.
- I have worked with public health data (surveillance, public health programs, etc.).
- I have worked with routine healthcare data.
- I attempted to use RWD data but gave up because of challenges.
- I have not worked with RWD.
- Other.

4. What software/tools do you use for:

      4a. RWD Collection (Case Report Forms or Data Collection Forms)?

      4b. Extracting Research Data from files provided by RWD source?

      4c. RWD Storage?

      4d. RWD Analysis?

**Your experience with data standards**

5. What is your experience with implementing CDISC or other standards? (mark all that apply)

- I have implemented CDISC standards for regulatory submissions.
- I have implemented CDISC standards outside of regulated clinical trials.
- I have reviewed data that uses CDISC standards as part of a regulatory submission process
- I have heard of CDISC standards while implementing other data standards.
- I have heard of CDISC standards but I have not implemented or reviewed CDISC standards or any other data standards before.
- I have never heard about CDISC standards until now
- Other:

6. If you have experience using CDISC standards, which of the following sources was most useful? (mark all that apply)

- Example case report forms
- Example data tables
- CDISC education courses
- CDISC webinars
- CDISC website
- CDISC conferences and working groups
- CDISC Standards documentation
- LinkedIn groups
- PhUSE conferences and working groups
- Volunteering on CDISC teams
- Other:

7. What were the key benefits of implementing CDISC standards?

8. What were the main challenges in implementing CDISC standards?

9. Did you implement CDISC standards for your own research or were they implemented at the institutional level and how?

SECTION 2 - Modified Qualitative Delphi

This section will be used for us to help the Advisory Board members debate around key concepts and identify agreements and differences to generate a common vision and strategy for CDISC RWD Connect. Your individual responses will be seen exclusively by the survey organizers. We will only provide consolidated anonymized information back to the Advisory Board members.

**Definition of RWD:**

As mentioned, for this initiative, we will use the following working definitions from the CDISC glossary, which are aligned with the FDA’s definitions:

- Real-world data (RWD): Data relating to patient health status and/or the delivery of health care routinely collected from sources other than traditional clinical trials. Examples of sources include data derived from electronic health records (EHRs); medical claims and billing data; data from product and disease registries; patient-generated data, including from in-home-use settings; and data gathered from other sources, such as mobile devices, that can inform health status.
- Real-world evidence (RWE): The clinical evidence derived from analysis of Real-World Data (RWD) regarding the usage and potential benefits or risks of a medical product.

10. What are your thoughts on the following two diagrams and where RWD fits in? Do you have any comments or suggestions for change or addition? Please support your comments with references whenever possible.


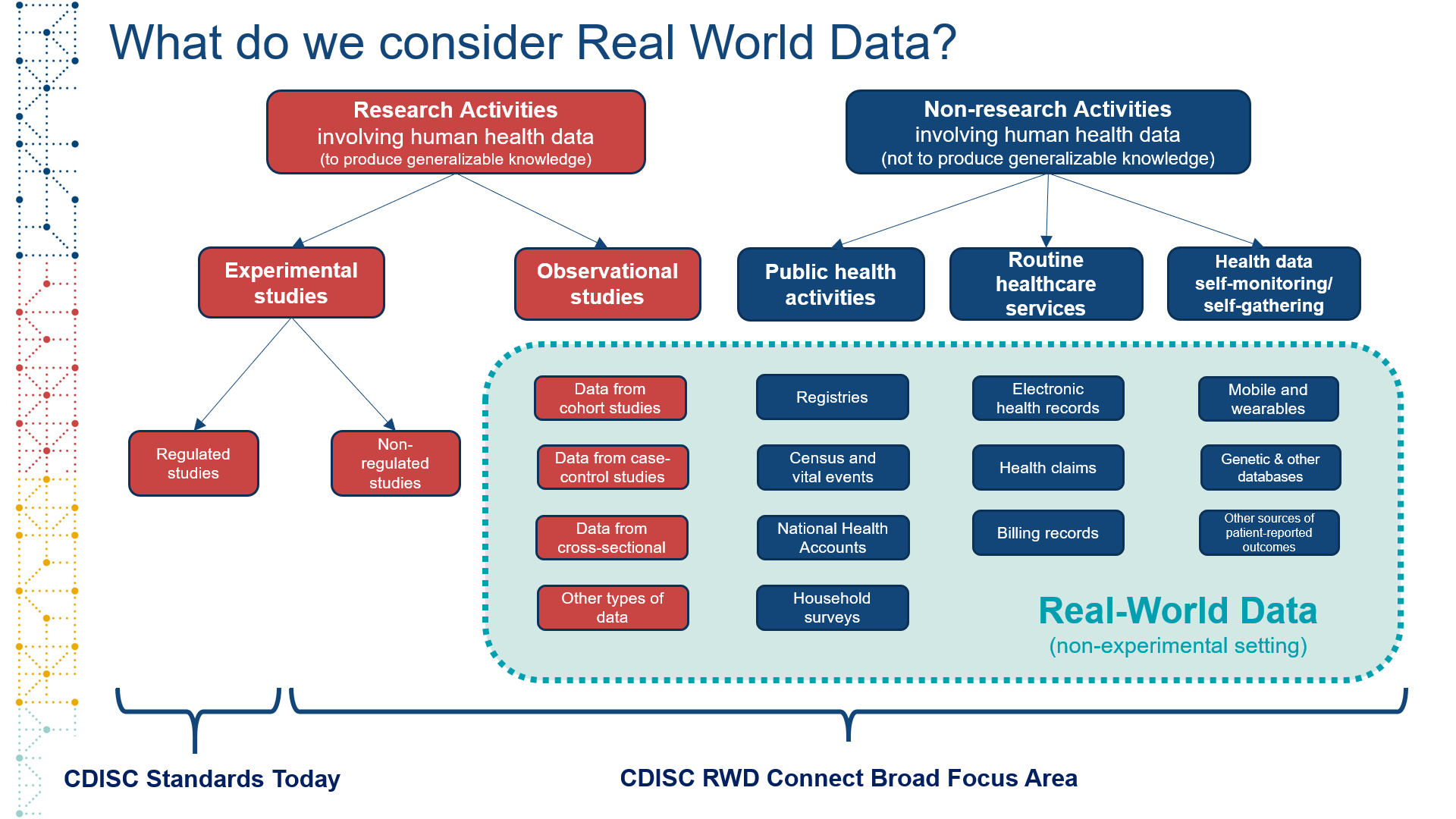


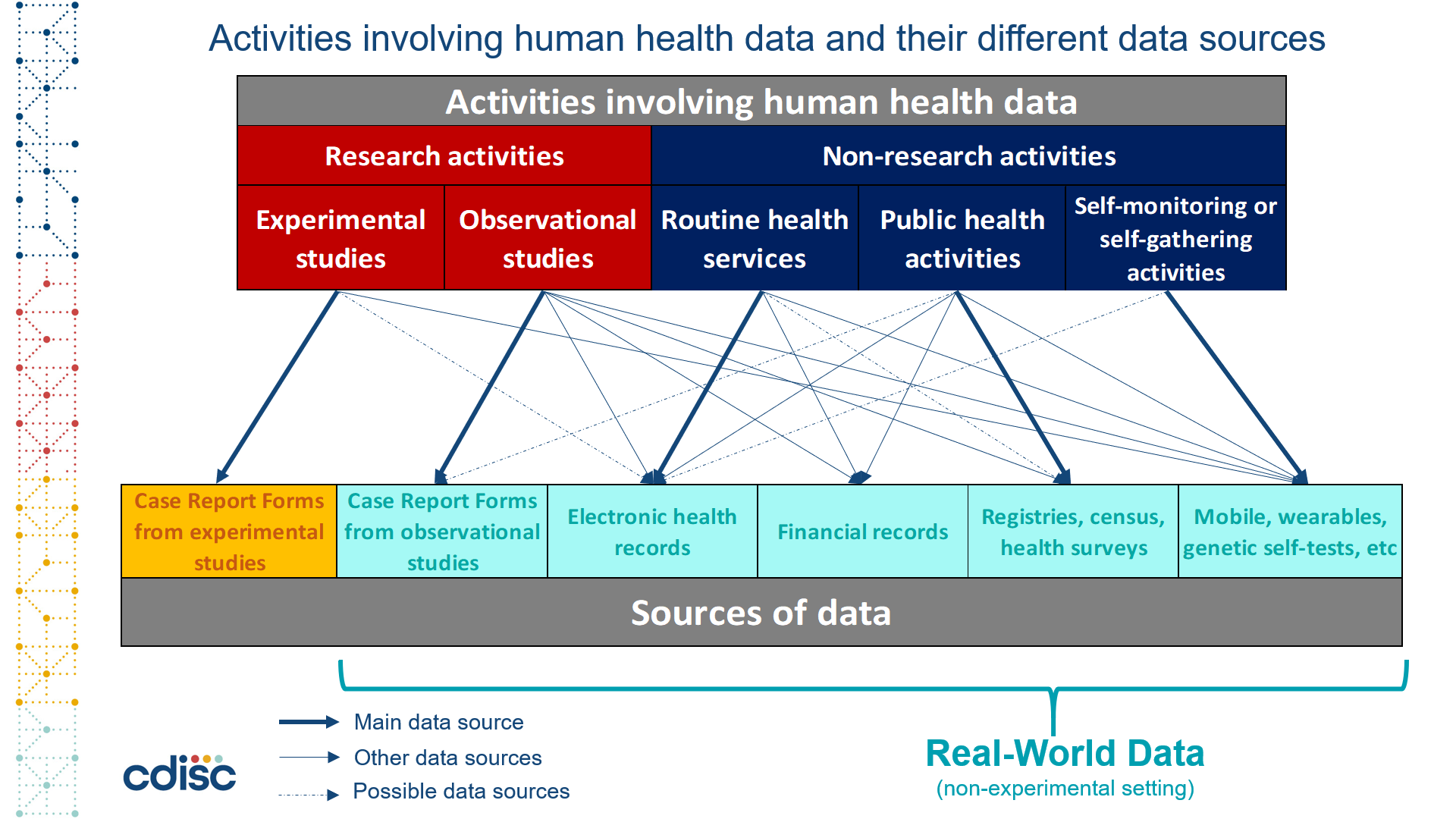


11. Which of the different components of RWD (as per the first diagram showed) do you think CDISC should focus on first, and why?

**Barriers to the use of data standards for RWD**

12. What are the most significant barriers to using CDISC data standards in academic settings for RWD?

13. What do you think it would take to achieve implementation of CDISC data standards across or within academic institutions?

14. Do you know of any efforts to use CDISC standards with RWD that have been published? If so, please share the links to the publications or supporting documents.

**Making the case for using data standards for RWD**

15. What do you see as the main challenges in academic clinical research that could be overcome with increased standardization of RWD?

16. What tools or support would help as RWD becomes more standardized?

17. What do you see as the primary benefits and opportunities from standardization of RWD? Specifically, how would you make this case to your colleagues?

18. What are the most effective ways to build knowledge and expertise on implementation of data standards in academic institutions? Please be specific.

19. What is the best way to reward or promote the use of data standards in academic settings?

**Devices and Wearables**

20. Are you aware of any data standards specifically for wearables or other medical device data?

21. What do you think are the most significant challenges related to implementing data standards for innovative data gathering technologies such as consumer wearables (e.g. Fitbit, Apple watches, cardio monitoring).

**Patient's Perspective in RWD**

22. How would you recommend that CDISC include the patient perspective in this initiative?

**The Future of RWD and CDISC Standards**

23. Forgetting for a moment about time and resource limitations, if you could wish for three things that could be done to make implementing CDISC standards for RWD easier, what would those three things be?

24. Which other data standards would be most important to consider collaborating/connecting with for this initiative and why?

25. Future state - What do you hope to be able to do in 3-5 years with regards to RWD standardization? Provide a ranking according to your priorities.

Share or aggregate routine healthcare data

- Utilize RWD as inputs for regulatory submissions
- Standardize multiple sources of RWD to generate meta-analysis
- Standardize multiple sources of RWD to consolidate them for other purposes (please describe)
- Share or aggregate patient level data from registries
- Standardize RWD for public health purposes
- Other:
