# Supplementary material for: Use of Clinical Data Interchange Standards Consortium (CDISC) Standards for Real-world Data: Expert Perspectives From a Qualitative Delphi Survey
Source: JMIR Med Inform. 2022 Jan 27;10(1):e30363. doi: 10.2196/30363 (PMC8832264; doi:10.2196/30363)
Supplement: Multimedia Appendix 2 [file medinform_v10i1e30363_app2.docx]

**Multimedia Appendix 2. Collaborations with other standards and initiatives.**

We asked EAB members what other data standards would be most important to consider collaborating/connecting with for this initiative and why. Participants recommended the following standards and collaborations:

| **Organization/Standard** | **Link** |
| --- | --- |
| ContSys | https://contsys.org/page/default |
| HL7 Fast Healthcare Interoperability Resources (FHIR) | https://www.hl7.org/fhir/ |
| HL7’s Clinical Information Models | https://www.hl7.org/Special/Committees/cimi/index.cfm |
| ICH Integrated Addendum to ICH E6 (R1) | https://www.fda.gov/files/drugs/published/E6%28R2%29-Good-Clinical-Practice--Integrated-Addendum-to-ICH-E6%28R1%29.pdf |
| ICHOM | https://www.ichom.org |
| ICSR E2B R2 and R3 | https://www.fda.gov/regulatory-information/search-fda-guidance-documents/e2br3-electronic-transmission-individual-case-safety-reports-implementation-guide-data-elements-and.  https://www.ema.europa.eu/en/documents/scientific-guideline/international-conference-harmonisation-technical-requirements-registration-pharmaceuticals-human-use_en-4.pdf |
| Institute of Electrical and Electronics Engineers (IEEE) | https://www.ieee.org |
| ISO IDMP | https://www.fda.gov/media/96181/download |
| ISO13606 | https://www.iso.org/standard/40784.html |
| Joint Initiative Council | http://www.jointinitiativecouncil.org |
| mCODE | https://mcodeinitiative.org |
| MedDRA | https:/www.meddra.org |
| National Database for Autism Research (NDAR) | https://nda.nih.gov/about.html |
| NCI Cancer Research Data Commons initiative - Center for Cancer Data Harmonization (CCDH) | https://datascience.cancer.gov/data-commons/center-cancer-data-harmonization-ccdh |
| OMOP Common Data Model | https://www.ichom.org |
| The Center for Expanded Data Annotation and Retrieval  (CEDAR) | https://www.ncbi.nlm.nih.gov/pmc/articles/PMC7098808/ |
| US National Institutes of Health (NIH) Common Data Elements (CDE) | https://www.nlm.nih.gov/cde/index.html |

Suggested terminologies:

| **Terminologies** | **Link** |
| --- | --- |
| ICD | https://www.cdc.gov/nchs/icd/index.htm |
| LOINC | https://loinc.org |
| MedDRA | https://www.meddra.org |
| NCI-EVS | https://evs.nci.nih.gov |
| NICHD | https://www.nichd.nih.gov |
| SNOMED CT | http://www.snomed.org |
| WHODrug Global | https://www.who-umc.org |

EAB members also recommended the following initiatives:

| **Initiative** | **Link** |
| --- | --- |
| COMET Initiative | https://www.comet-initiative.org |
| DICOM | https://www.dicomstandard.org |
| HL7 | http://www.hl7.org |
| Human Phenotype Ontologies (HPO) | https://hpo.jax.org/app/ |
| Orphanet Rare Disease ontology (ORDO) | http://www.orphadata.org/cgi-bin/index.php |
| PCORNET | https://pcornet.org |
| SENTINEL | https://www.fda.gov/safety/fdas-sentinel-initiative |
| USCORE | https://www.hl7.org/fhir/us/core/ |

The EAB also recommended collaborating with:

| **Organization** | **Link** |
| --- | --- |
| CONSORT for EHEALTH | https://pubmed.ncbi.nlm.nih.gov/23920638/ |
| The CTSA NCATS | https://ncats.nih.gov/ctsa |
| The US Office of the National Coordinator | https://www.healthit.gov |
