# Supplementary material for: Use of Clinical Data Interchange Standards Consortium (CDISC) Standards for Real-world Data: Expert Perspectives From a Qualitative Delphi Survey
Source: JMIR Med Inform. 2022 Jan 27;10(1):e30363. doi: 10.2196/30363 (PMC8832264; doi:10.2196/30363)
Supplement: Multimedia Appendix 3 [file medinform_v10i1e30363_app3.docx]

**Multimedia Appendix 3. List of expert advisory board members.**

| **Name** | **Affiliation** |
| --- | --- |
| Yuki Ando | Pharmaceutical and Medical Devices Agency (PMDA) |
| Yoshihiro Aoyagi | National Cancer Center Hospital East |
| Adam Asare | Director of IT for the UCSF Breast Care Clinic |
| Wenjun Bao | SAS Institute |
| Lisa Blackwell | Oxford University |
| Greg Botwin | Cedars-Sinai Medical Center, Los Angeles |
| Francesca Cerreta | European Medicines Agency |
| Ronald Cornet | Amsterdam University Medical Center, *The Netherlands* |
| Jacqueline Corrigan-Curay | Food and Drug Administration-Center for Drug Evaluation and Research (FDA-CDER) |
| Emilie Darcillon | Nestle |
| Susana Dodd | Liverpool University |
| Janel Fedler | Clinical Trials Statistical and Data Management Center (CTSDMC), University of Iowa |
| Matt Fisher | Clinical Trials Statistical and Data Management Center (CTSDMC), University of Iowa |
| Jason Gerson | Patient Centered Outcomes Research Institute (PCORI) |
| Gideon (Scott) Gordon | Food and Drug Administration-Center for Drug Evaluation and Research, Office of Strategic Programs (FDA-CDER-OSP) |
| Lorenzo Guizzaro | European Medicines Agency (EMA) |
| Karin Hedenmalm | European Medicines Agency (EMA) |
| Victoria Hedley | University of Newcastle |
| Ralf Herold | European Medicines Agency (EMA) |
| Shiro Hinotsu | Professor at the Department of Biostatistics at Sapporo Medical University, Sapporo University. Japan ARO Council. |
| Ingeborg Holt | IBM, Global Business Services |
| Lynn Hudson | The Critical Path Institute |
| Trevis Huff | Clinical Trials Statistical and Data Management Center (CTSDMC), University of Iowa |
| Georgina Humphries | The Wellcome Trust |
| Virginia Hussong | Food and Drug Administration-Center for Drug Evaluation and Research (FDA-CDER) |
| Masakatsu Imoto | Agency for Medical Research and Development (AMED) |
| Scott Kahn | The Lenora M. and Harry B. Helmsley Foundation |
| Matilde Kam | Food and Drug Administration-Center for Drug Evaluation and Research (FDA-CDER) |
| Qingna Li | Xiyuan Hospital, China Academy of Chinese Medical Sciences |
| Daisuke Koide | University of Tokyo, member of National University Hospital Council ARO TG3 |
| Sang Gyu Kwak | Daegu University Medical School, South Korea |
| Junkai Lai | Peking University Clinical Research Institute |
| Hanneke Lankheet | Danone |
| Becca Leary | University of Newcastle |
| Eri Matsuki | Agency for Medical Research and Development (AMED) |
| Carolina Mendoza-Puccini | National Institute of Neurological Disorders and Stroke  (NIH -NINDS) |
| Gong Mengchun | Digital Health China Technologies, Beijing, China  Institute of Health Management, Southern Medical University, Guangzhou, China |
| Laura Merson | Infectious Diseases Data Observatory (IDDO) |
| Erin Muhlbradt | National Institute of Health – National Cancer Institute – Enterprise Vocabulary Service (NIH-NCI-EVS) |
| Marianne Munene | Kenya Medical Research Institute (KEMRI) - Wellcome Trust Kenya |
| Lillian Mwango | Kenya Medical Research Institute (KEMRI) - Wellcome Trust Kenya |
| Harumasa Nakamura | Institute of Genetic Medicine, Newcastle University, Newcastle upon Tyne, UK. National Center of Neurology and Psychiatry, Tokyo, Japan |
| Mihoko Okada | Institute of Health Data Infrastructure for All (IDIAL) Japan |
| Frank Pétavy | European Medicines Agency (EMA) |
| Vaishali Popat | Food and Drug Administration-Center for Drug Evaluation and Research (FDA-CDER) |
| Kenneth Quinto | Food and Drug Administration-Center for Drug Evaluation and Research (FDA-CDER) |
| Christina Reith | Oxford University |
| Lyubov Remennik | National Institute of Health- National Cancer Institute – Enterprise Vocabulary Service (NIH-NCI-EVS) |
| Mitra Rocca | Food and Drug Administration – Office of Computational Science (FDA-OCS) |
| Frank Rockhold | Duke Clinical Research Institute; Duke University |
| Yaoping Ruan | LinkDoc technology, Beijing |
| Toshiki I. Saito | National Hospital Organization (NHO) Nagoya Medical Center (NMC) |
| Maria Shin | Daegu Catholic University Medical Center, South Korea |
| Erin Sizemore | Centers for Disease Control |
| Enti Spata | Oxford University |
| Will Stevens | Oxford University |
| Catrin Tudur-Smith | Liverpool University |
| Satoshi Ueno | National Institute of Public Health Japan |
| Sam Volchenboum | University of Chicago |
| Naomi Waithira | MORU, Bangkok, Thailand |
| Anita Walden | University of Arkansas, Medical Sciences, HL7 |
| Jason Walker | Harvard Medical School, Massachusetts General Hospital |
| Karl Wallendszus | Oxford University |
| Denise Warzel | National Institute of Health – National Cancer Institute (NIH-NCI) |
| Jon Yankey | Clinical Trials Statistical and Data Management Center (CTSDMC), University of Iowa |
| Chen Yao | Peking University Clinical Research Institute |
| Meredith Zozus | The University of Texas Health Sciences Center, Houston, HL7 |
