# Supplementary material for: Use of Clinical Data Interchange Standards Consortium (CDISC) Standards for Real-world Data: Expert Perspectives From a Qualitative Delphi Survey
Source: JMIR Med Inform. 2022 Jan 27;10(1):e30363. doi: 10.2196/30363 (PMC8832264; doi:10.2196/30363)
Supplement: Multimedia Appendix 4 [file medinform_v10i1e30363_app4.docx]

**Multimedia Appendix 4. Delphi questionnaire.**

CDISC RWD Connect – Qualitative Delphi Survey: First Round

SECTION 1 - Questions for background information

This section is intended to provide a better understanding of the participants' knowledge and experience in different areas relevant to the CDISC RWD Connect Initiative. Your individual responses will be seen exclusively by the organizers. We will only provide consolidated anonymized information back to the Advisory Board members.

1. First and last names of the people who contributed responses to this survey.

1. Relevant institution/s you are affiliated with (mark all that apply and specify institution name)

• University

• Research center

• Non-profit organization

• Government organization

• International organization

• Other:

**Your experience with Real-World Data (RWD)**

While there are multiple definitions of real-world data (RWD) currently in use, the CDISC Glossary has adopted the following definition, which is aligned with the FDA's definition: “*Data relating to patient health status and/or the delivery of health care routinely collected from sources other than traditional clinical trials. Examples of sources include data derived from electronic health records (EHRs); medical claims and billing data; data from product and disease registries; patient-generated data, including from in-home-use settings; and data gathered from other sources that can inform on health status, such as mobile devices.”*

With this definition in mind, the next section is focused on capturing your experience using RWD.

3. What is your experience using RWD? (mark all that apply)

- I have conducted experimental research/academic studies using RWD that were not intended for regulatory submission. (when people click this option, a follow-up question should open to ask the respondent "Please describe:")
- I have conducted observational research studies (cohort study, case-control, etc.). (when people click this option, a follow-up question should open to ask the respondent "Please describe:")
- I have worked with multiple RWD sources to conduct research around healthcare delivery. (when people click this option, a follow-up question should open to ask the respondent "Please describe:")
- I have worked with public health data (surveillance, public health programs, etc.). (when people click this option, a follow-up question should open to ask the respondent "Please describe:")
- I have worked with routine healthcare data. (when people click this option, a follow-up question should open to ask the respondent "Please describe:")
- I attempted to use RWD data, but gave up because of challenges. (when people click this option, a follow-up question should open to ask the respondent "Please describe:")
- I have not worked with RWD.
- Other.

4. What software/tools do you use for:

4a. RWD Collection (Case Report Forms or Data Collection Forms)?

4b. Extracting Research Data from files provided by RWD source?

4c. RWD Storage?

4d. RWD Analysis?

**Your experience with data standards**

5. What is your experience with implementing CDISC or other standards? (mark all that apply)

- I have implemented CDISC standards for regulatory submissions.
- I have implemented CDISC standards outside of regulated clinical trials. (when people click this option, a follow-up question should open to ask the respondent "Please describe:")
- I have reviewed data that uses CDISC standards as part of a regulatory submission process.
- I have heard of CDISC standards while implementing other data standards. (when people click this option, a follow-up question should open to ask the respondent "Please describe:")
- I have heard of CDISC standards, but have not implemented or reviewed them or any other data standards before.
- I have never heard of CDISC standards until now. (when people click this option, the survey should end for them.)
- Other:

(questions 6 to 9 should only activate for respondents who marked either of the first two options of question 5)

6. If you have experience using CDISC standards, which of the following sources was most useful? (mark all that apply)

- Example case report forms
- Example data tables
- CDISC education courses
- CDISC webinars
- CDISC website
- CDISC conferences and working groups
- CDISC Standards documentation
- LinkedIn groups
- PHUSE conferences and working groups
- Volunteering on CDISC teams
- Other:

7. What were the key benefits of implementing CDISC standards?

8. What were the main challenges in implementing CDISC standards?

9. Did you implement CDISC standards for your own research or were they implemented at the institutional level and how?

SECTION 2 - Modified Qualitative Delphi

This section will be used for us to help Advisory Board members discuss key concepts and identify agreements and differences to generate a common vision and strategy for CDISC RWD Connect. Your individual responses will be seen exclusively by the survey organizers. We will only provide consolidated anonymized information back to the Advisory Board members.

**Definition of RWD:**

As mentioned for this initiative, we will use the following working definitions from the CDISC glossary, which are aligned with the FDA’s definitions:

Real-world data (RWD): Data relating to patient health status and/or the delivery of health care routinely collected from sources other than traditional clinical trials. Examples of sources include data derived from electronic health records (EHRs); medical claims and billing data; data from product and disease registries; patient-generated data, including from in-home-use settings; and data gathered from other sources, such as mobile devices, that can inform health status.

Real-world evidence (RWE): The clinical evidence derived from analysis of Real-World Data (RWD) regarding the usage and potential benefits or risks of a medical product.

10. What are your thoughts on the following two diagrams and where RWD fits in? Do you have any comments or suggestions for change or addition? Please support your comments with references whenever possible.

11. Which of the different components of RWD (as per the first diagram showed) do you think CDISC should focus on first, and why?

**Barriers to the use of CDISC standards for RWD**

12. What are the most significant barriers to using CDISC standards in academic settings for RWD?

13. What do you think it would take to achieve implementation of CDISC standards across or within academic institutions?

14. Do you know of any efforts to use CDISC standards with RWD that have been published? If so, please share the links to the publications or supporting documents.

**Making the case for using CDISC standards for RWD**

15. What do you see as the main challenges in academic clinical research that could be overcome with increased standardization of RWD?

16. What tools or support would help as RWD becomes more standardized?

17. What do you see as the primary benefits and opportunities from standardization of RWD? Specifically, how would you make this case to your colleagues?

18. What are the most effective ways to build knowledge and expertise on implementation of CDISC standards in academic institutions? Please be specific.

19. What is the best way to reward or promote the use of CDISC standards in academic settings?

**Devices and Wearables**

20. Are you aware of any data standards specifically for wearables or other medical device data?

21. What do you think are the most significant challenges related to implementing data standards for innovative data gathering technologies such as consumer wearables (e.g. Fitbit, Apple watches, cardio monitoring).

**Patient's Perspective in RWD**

22. How would you recommend that CDISC include the patient perspective in this initiative?

**The Future of RWD and CDISC Standards**

23. Forgetting for a moment about time and resource limitations, if you could wish for three things that could be done to make implementing CDISC standards for RWD easier, what would those three things be?

24. Which other data standards would be most important to consider collaborating/connecting with for this initiative and why?

25. Future state - What do you hope to be able to do in 3 - 5 years with regard to RWD standardization? Provide a ranking according to your priorities. (people should be able to rank the options below)

- Share or aggregate routine healthcare data.
- Utilize RWD as inputs for regulatory submissions.
- Standardize multiple sources of RWD to generate meta-analysis.
- Standardize multiple sources of RWD to consolidate them for other purposes (please describe).
- Share or aggregate patient level data from registries.
- Standardize RWD for public health purposes.
- Other:

Thank you for your thoughtful completion of this survey!
